# Assessing Benzene Dimer Interactions in Solution With a Molecular Balance

**DOI:** 10.1002/chem.71126

**Published:** 2026-05-18

**Authors:** Marvin H. J. Domanski, Michael Fuhrmann, Nils F. Hacket, Heike Hausmann, Peter R. Schreiner

**Affiliations:** ^1^ Institute of Organic Chemistry Justus Liebig University Giessen Germany

**Keywords:** CH‐π Interactions, London dispersion, noncovalent interactions, solvent effects, steric effects

## Abstract

We present a combined experimental and computational study of a 1,4/1,6‐dibenzyl substituted cyclooctatetraene (COT)‐based molecular balance as a model to assess the benzene dimer interactions in solution. This is accomplished by investigating the equilibrium between the unfolded 1,4‐ versus folded 1,6‐dibenzyl COT in a variety of common organic solvents. NMR measurements demonstrate that the 1,6‐COT isomer, in which the phenyl moieties are in proximity, is always favored over the unfolded and sterically less hindered 1,4‐COT by ∼0.3 kcal mol^−1^, irrespective of solvent. Computations identify London dispersion (LD) interactions between the aromatic moieties in 1,6‐COT as the main stabilizing force.

## Graphical abstract

The Gecko in the ToC was generated with Canva (31.03.2025 11:00 pm), the remaining graphics by the authors.

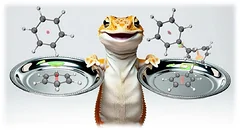

## 1 Introduction

Noncovalent interactions (NCIs) between aromatic moieties play a key role in biochemistry [1], material science [2], catalysis [3, 4, 5, 6, 7, 8, 9, 10, 11], and provide important insights into reaction mechanisms as well as selectivities, as exemplified by the previously investigated Johnson−Corey−Chaykovsky epoxidation [12]. These interactions help rationalize protein folding [13, 14, 15, 16], stacking of DNA bases [17], or enantioselectivity in catalytic transformations [5, 10]. Chemists often refer to NCIs between aromatic moieties as “π‐π” stacking or “σ‐π” interactions (amongst other names) [2, 18, 19]. However, multiple detailed studies on, for example, the benzene dimer conclude that the π‐orbitals do not significantly engage in quadrupole overlap‐driven bonding and the benzene dimer is best described as a van der Waals complex in which charge transfer, induction, exchange‐repulsion, and primarily London dispersion (LD) interactions play a dominant role with an R^−6^ distance dependence of the dimerization energy [18, 20, 21]. These findings have replaced the originally proposed model and rules suggested by Hunter et al. [17, 22, 23], who emphasized the quadrupole‐quadrupole electrostatic component of the stacking interaction.

**FIGURE 1:**
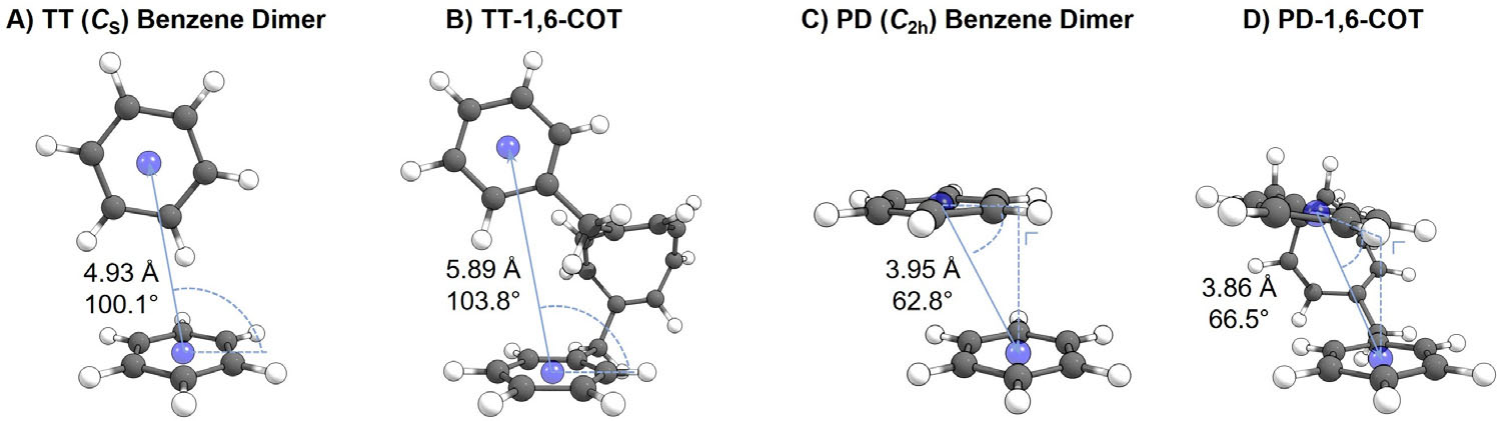
Left: The benzene dimer in the tilted T‐shaped (**TT**, *C*_s_ symmetry). Right: Parallel displaced (**PD**, *C*_2h_ symmetry) computed at the CCSD(T)/CBS level of theory [24]. In comparison, our **COT**‐model system to mimic the benzene dimer interactions in solution at the ωB97X‐D4/def2‐TZVPP level of theory. Ph‐Ph angles and distances are given for the center of each benzene to highlight the similarities between the reference and model system.

High‐level computational studies revealed that the two minima on the potential energy surface (PES) correspond to the tilted T‐shaped (**TT**, *C*_s_ symmetry) and parallel displaced (**PD**, *C*_2h_ symmetry) structure (Figure 1) [24]. Early studies suggested the *C*_2v_‐symmetric **T**‐shaped configuration as the global minimum. However, multiple sources now established that the tilted *C*_s_‐symmetric **T**‐shaped configuration (**TT**) is slightly more stable, with a dissociation energy of ∆*E* = 2.83 kcal mol^−1^ [24]. The **PD** dimer is the second minimum with a dissociation energy of ∆*E* = 2.68 kcal mol^−1^ [24, 25]. The computed values align well with gas‐phase ionization experiments, which have yielded dissociation energies of ∆*D*_0_ = 1.61 kcal mol^−1^ and 2.40 kcal mol^−1^ for benzene dimer formation [26, 27, 28].

However, interactions observed in the gas phase may not directly translate to solution, where entropic and solvent effects often weaken NCIs [7, 10, 29]. Consequently, the dissociation energies reported for benzene dimers in the gas phase may overestimate interaction strengths in solution.

To address this, the Wilcox group conducted pioneering work on **T**‐oriented aromatic interactions using molecular torsion balances [30]. These balances derive relative strengths of NCIs through equilibrium studies involving a “folded” (sterically hindered) state, where two moieties interact intramolecularly, and an “unfolded” (sterically less hindered) state, where the groups are separated and their interactions minimized [31]. ^1^H‐NMR spectroscopy revealed that the **T**‐shaped arrangement of the aromatic moieties in the Wilcox balance exhibited a Gibbs free interaction energy of 0.24 kcal mol^−1^ in CDCl_3_ at 298 K, significantly smaller than the gas‐phase values for the benzene dimer [24, 26, 30]. Subsequently, Shimizu and coworkers explored aromatic stacking interactions using *N*‐arylimide‐based molecular balances, where aromatic moieties interact in a **PD**‐like configuration [32, 33]. They reported that increasing the size of the aromatic systems did not lead to significantly stronger interactions, concluding that the LD contribution to stabilization is small in solution [32, 33].

Later, Cockroft extended this research by studying polyaromatic systems with molecular balances, showing that the interaction energy cannot be explained solely by size of interacting groups [34]. Instead, the interaction energy correlates with the solvent‐accessible surface area (SASA), which describes the area of solvent‐solute interactions, of the folded and unfolded conformers. Additionally, high solvent bulk polarizability such as found for CS_2_ exhibit the least favorable stacking interactions due to competing LD interactions between solvent and solute [24, 34].

All molecular balances have in common that the observed interaction energies in solution are significantly weaker than those in the gas phase. Chen and coworkers demonstrated that NCIs can be attenuated by up to 80% in aprotic solvents compared to the corresponding gas‐phase structures, based on studies of proton‐bound dimers [35, 36]. Subsequently, Shimizu and coworkers revisited this topic using rigid CH–π molecular balances. They observed that enlarging the π‐system, thereby enhancing dispersion interactions, indeed increases the interaction strength. This stabilization is again substantially reduced, by as much as 80% in solution [37].

**FIGURE 2:**
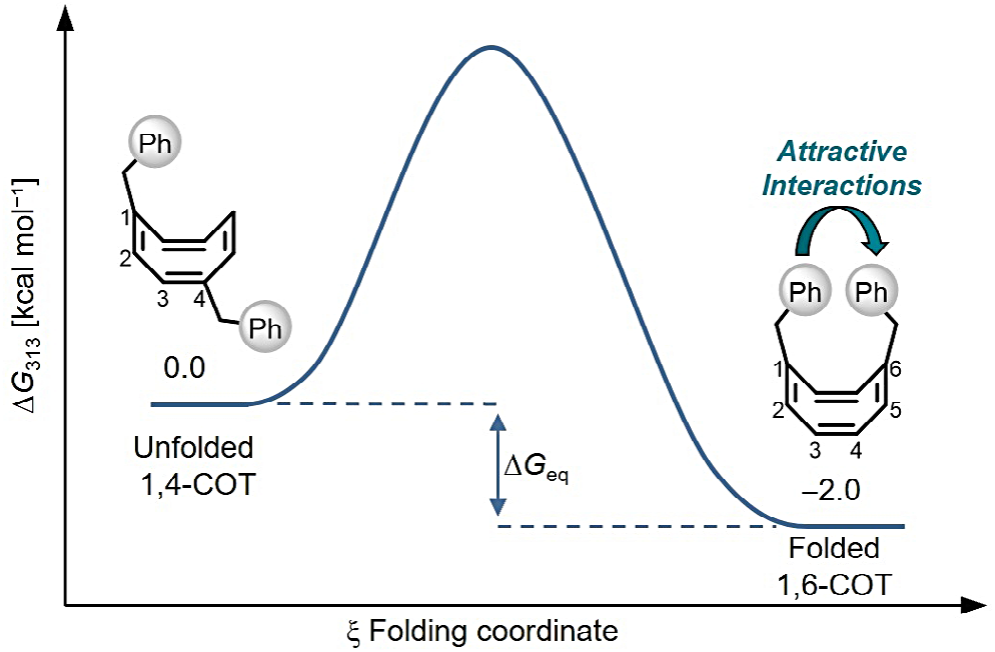
Schematic energy profile of the equilibrium between 1,4/1,6‐COT. Gibbs free energies (313 K) are given at the ωB97X‐D4/def2‐TZVPP [46, 47] level of theory in the gas phase.

However, owing to the incorporation of polar groups of these model systems the benzene dimer interactions may be affected by local dipole interactions that also alter the local interactions with the solvent. To address these limitations, nonpolar molecular hydrocarbon balances, such as the **1,4/1,6** substituted cyclooctatetraene (**COT**), have been developed [29, 38]. The **1,4‐** and **1,6‐COT** diastereomers are valence bond isomers that interconvert via a planar π‐bond isomerization. In **1,4‐COT** the substituents are separated from each other, while **1,6‐COT** brings them in close proximity and allows them to interact (Figure 2) [29]. The combination of high symmetry, absence of bond dipoles, temperature‐sensitive and well‐resolved NMR signals makes them ideally suited for quantifying small differences in intramolecular interaction energies. Previous studies revealed that intramolecular LD interactions do not cancel in solution and are in fact quite constant over a large range of solvent polarities and polarizabilities [29, 38]. These findings were further supported by **COT**‐based molecular balances functionalized with highly polarizable alkyl [29, 38, 39, 40, 41] and silyl [42, 43] moieties often referred to as dispersion energy donors (DEDs) [7, 8, 44, 45].

## 2 Results and Discussion

Here we employ a dibenzyl‐substituted **COT** system in which the phenyl groups are connected to the **COT** backbone with a methylene spacer that allows the phenyl groups in the **1,6‐COT** isomer to adopt multiple conformations and therefore capture all possible interactions of the benzene dimer. We began with investigating the energetic landscape of the **1,4‐/1,6‐COT** system by computing all possible conformers of each isomer using CREST [48] and further optimized them at the ωB97X‐D4/def2‐TZVPP level of theory [46, 47]. Unfolded **1,4‐COT** shows no interaction between the phenyl moieties as they are separated by ∼8 Å, and this represents a model of the dissociated benzene dimer, which is ∆*G*_313 K_ = 2.0 kcal mol^−1^ higher in energy than **1,6‐COT**.

**FIGURE 3:**
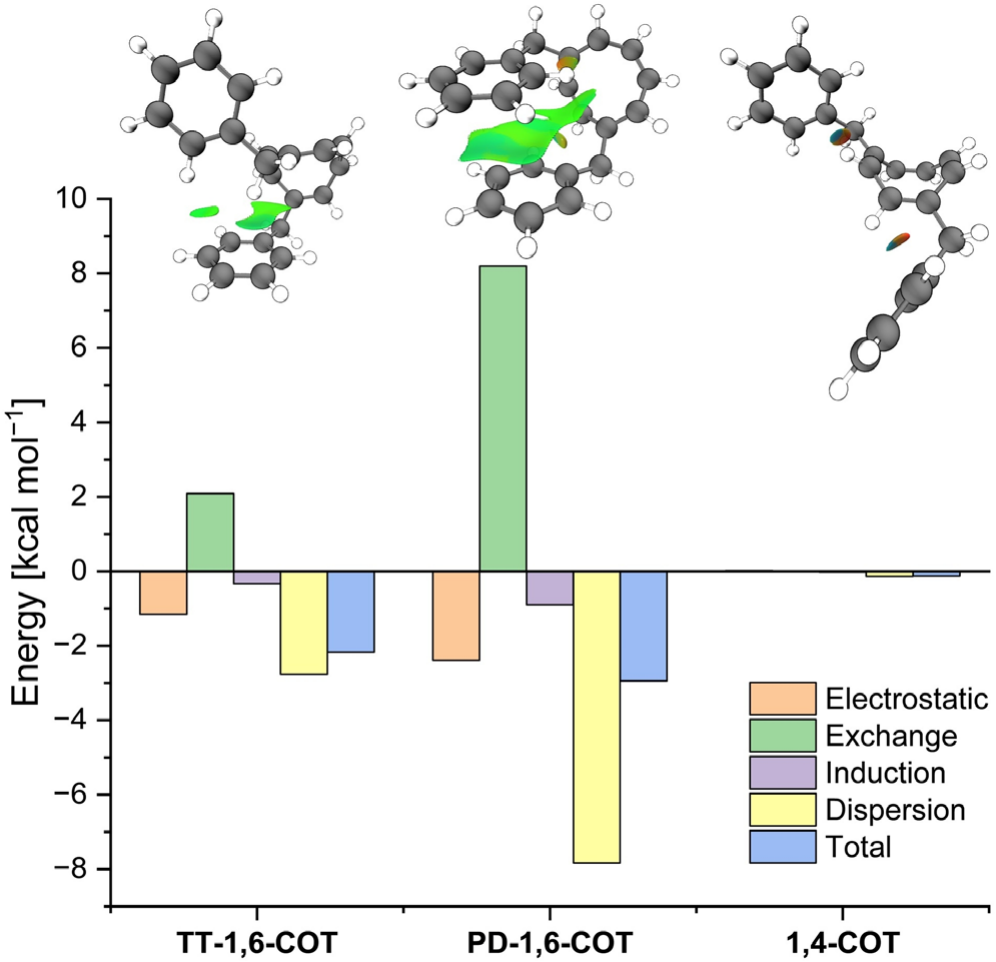
NCI plots [50] of **TT‐1,6‐COT** (left) and **PD‐1,6‐COT** (middle) as well as the unfolded **1,4‐COT** (right). Isosurfaces (isovalues sign(*λ*_2_)ρ = –0.04 a.u. to +0.04 a.u.) are color‐coded red (corresponding to strong repulsion) blue (strong attractive interactions), and green (weak noncovalent interactions). ISAPT [51, 52] computations were carried out using the SIAO1 [53] partition scheme by separating the substituents as well as the **COT**‐backbone into one fragment each and computing the interaction energy between the substituents at the fisapt0 jun‐cc‐pVDZ level of theory.

The two most stable conformers of **1,6‐COT** adopt **TT**‐ or **PD**‐shaped arrangements that are remarkably similar to the gas‐phase benzene dimer structures (Figure 1). **TT‐1,6‐COT** features a central phenyl distance of 5.89 Å and a slip angle of *θ*_A_ = 103.8°. In comparison, the **TT** benzene dimer has a shorter central phenyl‐phenyl distance of 4.93 Å and a slip angle of 100.1° (Figure 1) [24]. This results in slightly weaker interaction energies. However, the total SAPT energy between the substituents in our system is with 2.2 kcal mol^−1^ (Figure 3) close to the computed **TT‐**benzene dimer [49]. In **PD‐1,6‐COT**, the phenyl moieties have a distance of 3.86 Å and an angle of 66.5°. The phenyl group orientations in **TT‐** and **PD‐1,6‐COT** hence closely resemble the two lowest‐lying benzene dimer structures. DFT computations further reveal that **TT‐1,6‐COT** is slightly lower in energy than **PD‐1,6‐COT** (Table S14), resulting in a higher equilibrium population of **TT‐1,6‐COT**. Consequently, **1,4‐/1,6‐COT** model system can be employed to estimate the benzene dimer interactions in solution.

To examine the stabilizing NCIs in **1,6‐COT**, we took the ωB97X‐D4/def2‐TZVPP [46, 47] optimized structures and performed an NCI plot analysis [50] (Figure 3). In **TT‐1,6‐COT**, stabilizing NCIs are present both between the two phenyl groups and between the phenyl group and the CH_2_ spacer unit. In contrast, in **PD‐1,6‐COT**, these interactions are primarily driven by the interaction between the two phenyl groups. To quantify and dissect the NCIs into physically meaningful components, we performed ISAPT [51, 52] computations using the SIAO1 [53] partition scheme by separating the substituents and the **COT**‐backbone into one fragment each and computing the interaction energies between the substituents (Figure 3). For **TT‐1,6‐COT** and **PD‐1,6‐COT** inductive effects only play a minor role, electrostatic and mainly LD stabilize the dimers and compensate Pauli repulsion, resulting in a net negative total energy, in agreement with benzene and toluene dimer computations [54]. These results are also supported by a local energy decomposition (LED) analysis (reported in the SI, including the **1,4‐COT**) [55, 56]. The total SAPT‐interaction energy in the **TT‐1,6‐COT** accounts for 2.7 kcal mol^−1^ and is in very good agreement with the computed gas phase benzene dimer energy of 2.8 kcal mol^−1^ in the TT‐conformation. We computed the total interaction energy for **PD‐1,6‐COT** of 3.1 kcal mol^−1^ which also aligns well with the 2.7 kcal mol^−1^ obtained in recent computations for the PD‐benzene dimer [24].

**FIGURE 4:**
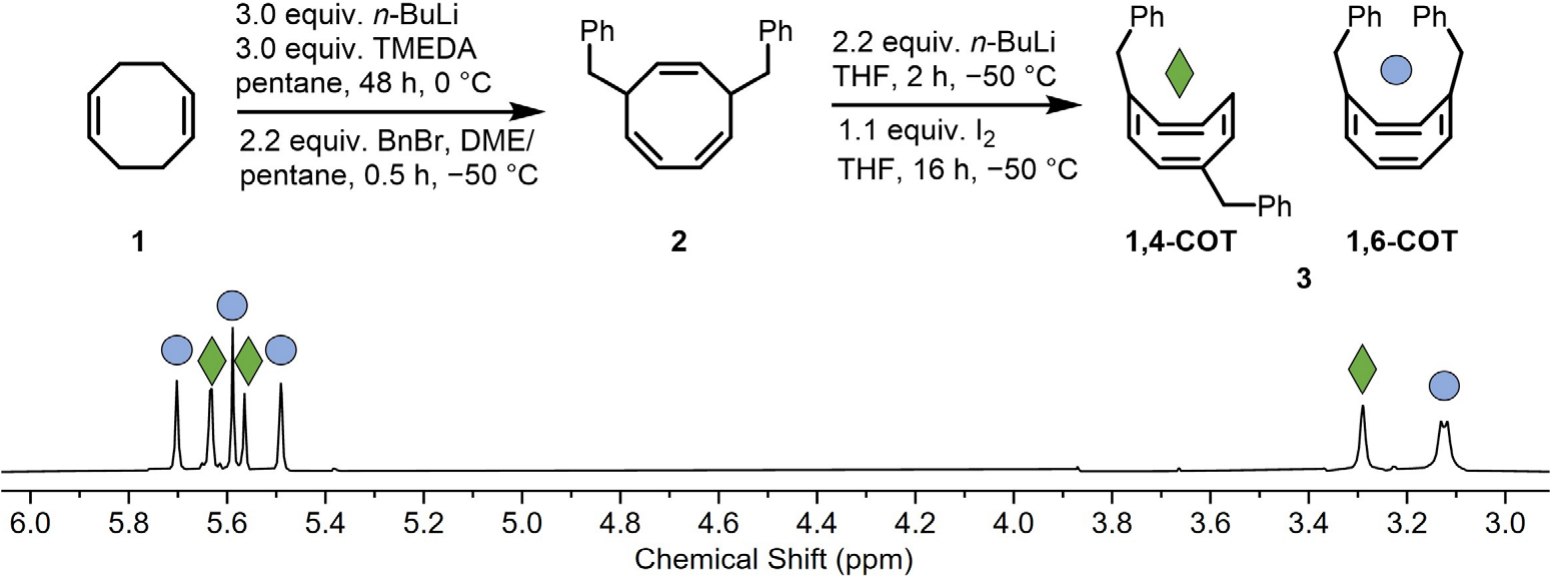
Synthesis of the target compounds starting from 1,5‐cyclooctadiene and ^1^H‐NMR assignments of **1,6‐COT** and **1,6‐COT** in DCE‐d_4_. Benzylic protons were used for integration and determination of the diastereomer ratios.

For the preparation of the experimental system, we adapted established literature procedures beginning with 1,5‐cyclooctadiene **1** to synthesize **1,4‐/1,6‐COT** (Figure 4) [41, 42, 57, 58, 59]. Lithiation of **1** with *n*‐BuLi gives the dianion that was trapped with benzyl bromide to provide triene **2**. Notably, we were able to isolate triene **2**, which, unlike its parent that decomposes rapidly, can be stored for several days at low temperatures. We oxidized **2** with iodine in the presence of *n*‐BuLi, yielding a mixture of **1,4/1,6‐COT**. Reversed‐phase HPLC purification gave pure **1,4/1,6‐COT**, with a total yield of 22% over two steps. The benzylic protons were assigned according to literature data and by comparing their proton shifts with the computed spectrum (see Supporting Information for details of all spectroscopic assignments and rate determinations in Figures S1–S18 and Tables S1–S12). All COT‐protons were assigned using 2D‐NMR techniques. The benzylic protons were used for the integration and determination of the isomer ratios. For computed NMR spectra for comparison, see Figure S25, S26.

To establish a suitable equilibration temperature for the isomer mixture, we warmed the samples gradually from 25 °C to 75 °C in 5 °C increments, holding each temperature for one hour in DCE‐*d*_4_. The recorded van't Hoff data revealed a linear relationship above 35 °C indicating that the equilibrium was reached within the equilibration time. At lower temperatures, the equilibration between **1,4/1,6‐COT** did not occur in a feasible time frame. Additionally, we conducted a series of experiments with varying concentrations of **1,4/1,6‐COT**, from 6 to 60 mM, and found no change in the isomer ratios, indicating concentration independence. All further measurements were carried out at 40 °C, with concentrations of 30 mM.

We measured an enthalpic contribution of ∆*H*  =  −0.52 ± 0.03 kcal mol^−1^ and an entropy contribution of ∆*S*  =  −0.722 ± 0.081 cal mol^−1^ K^−1^ (see Supporting Information for details), resulting in an *exergonic* equilibrium in favor of folded **1,6‐COT**; our ∆*G* = –0.30 ± 0.09 kcal mol^–^^1^ compares favorably with Wilcox’ –0.24 kcal mol^−1^ [30]. These findings are also in line with molecular balances functionalized with DEDs such as *^t^* Bu (∆*G* = –0.17± 0.01 kcal mol^−1^) [29, 38], adamantyl (∆*G* = –0.15 ± 0.01 kcal mol^–^^1^) [38], and diamantyl (∆*G* = –0.25±0.01 kcal mol^–^^1^) [38] in *n*‐octane at 298 K. As observed in these closely related systems, different solvents change the ∆*H* and ∆*S* values only slightly but typically in opposite directions (enthalpy‐entropy compensation) [29]. An intermediate conclusion is that benzene is one of the strongest mutual DED groups, which underscores its manifold use and recognition as enabling some key interactions highly relevant to chemical and biological systems (*vide supra*).

**FIGURE 5:**
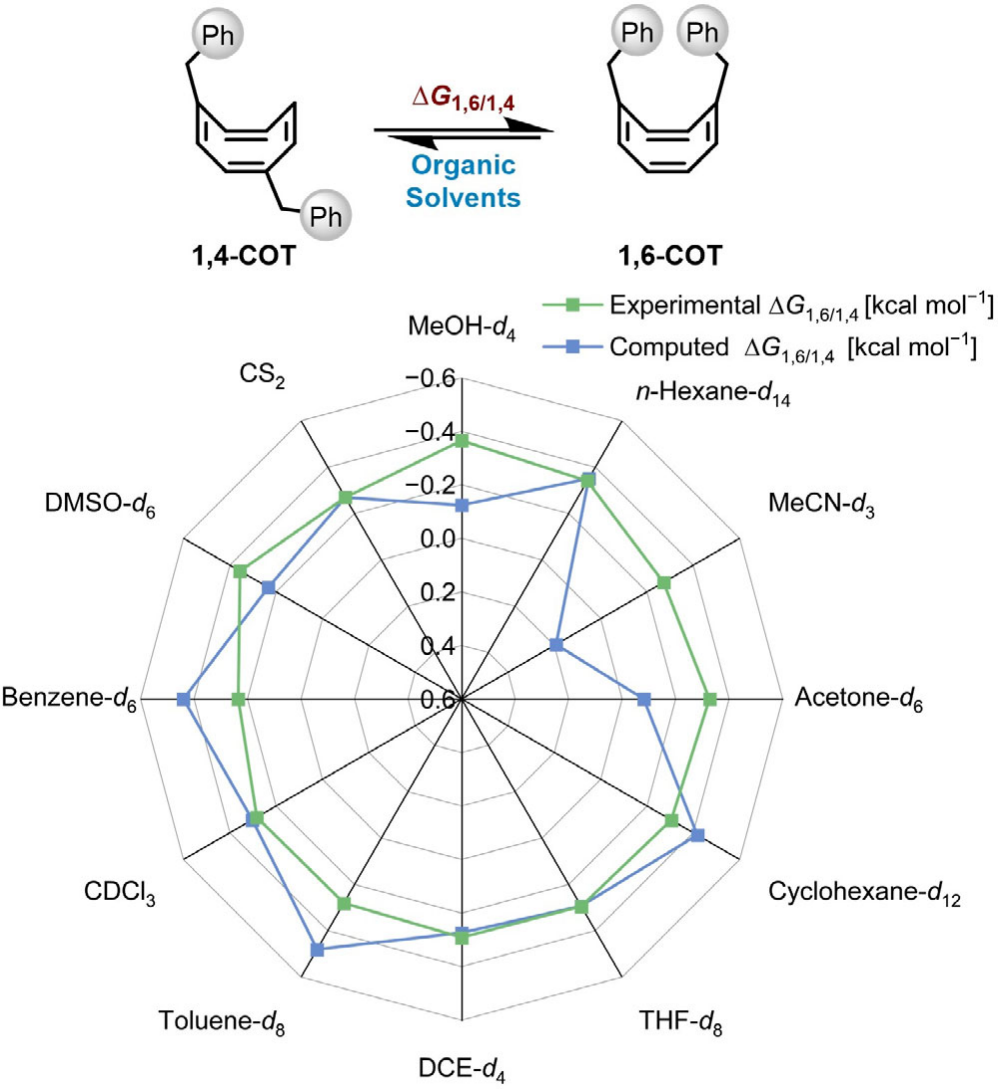
Experimentally (green) and computed (blue) ∆*G* values at 313 K for the **1,6/1,4‐COT** equilibrium ordered clockwise with respect to increasing solvent polarizabilities [61]. The substrate concentration for all measurements was 30 mM. Negative values correspond to an equilibrium favoring **1,6‐COT**. Computations were performed at ωb97X‐d4/def2‐TZVP[SMD]+GmRRHO(GFN2[alpb]‐bhess)//r2scan‐3c[SMD], taking all conformers for each isomer into account with a Boltzmann weighted distribution using CENSO [60].

To investigate solvent effects and their impact on the **1,4/1,6‐COT** equilibrium we further optimized the conformers of each isomer at the ωb97X‐D4/def2‐TZVP[SMD]+GmRRHO(GFN2[alpb]‐bhess)//r2scan‐3c[SMD], level of theory and applied a Boltzmann weighted distribution using CENSO [60] in twelve common organic solvents to correlate our findings across a range of solvent parameters. The computations predict that **1,6‐COT** is favored by 0.2–0.4 kcal mol^−1^ in all solvents (Figure 5, blue line; for details see Table S13–S17). This is in line with the solvent attenuation studies by Chen et al. [35, 36] with respect to the gas phase energies (*vide supra*). The only exception being acetonitrile in which **1,4‐COT** is predicted to be more stable; the reasons for this outlier are unclear at this time.

The experimental results confirm our computational predictions and show that the **1,6‐COT** is preferred over the **1,4‐COT** in all solvents and that the ratio remains remarkably constant (Figure 5, green line). The lowest **1,6/1,4‐COT** ratio was observed in benzene‐*d*_6_ (*K*_eq(1,6/1,4)_ = 1.46, ∆*G*_(1,6/1,4)_  =  −0.23 kcal mol^−1^), suggesting competitive interactions between solvent and solute; this has also been found for other balances. The **1,6‐COT** preference in hydrocarbon solvents follows the series benzene < toluene < cyclohexane < *n*‐hexane with decreasing solvent polarizability (SP) as well as decreasing cohesive energy density (ced) [61]. The highest ratio in favor of folded **1,6‐COT** was observed for the least polarizable solvent methanol‐*d*_4_ (*K*_eq(1,6/1,4)_  =  1.83, ∆*G*_(1,6/1,4) _ =  −0.37 kcal mol^−1^).

The reduced solvent accessible surface area of **1,6‐COT** (482.5 Å^2^) compared to **1,4‐COT** (573.6 Å^2^) reduces unfavorable solvent‐solute interactions in less polarizable solvents. The minimized molecular volume leads to a smaller cavity within the solvent cage, which additionally entropically favors **1,6‐COT**. Overall, there seems to be little to no correlation between the observed ratios and commonly employed solvent parameter scales such as SP, solvent polarity−polarizability (SPP) [61], or bulk SP [34] possibly owning to the absence of polar functional groups in our system. This finding highlights that intramolecular LD interactions between aromatic moieties do not change much over a large range of rather different solvents.

## 3 Conclusions

Here we present a **1,4‐/1,6**‐dibenzyl‐substituted **COT**‐based molecular balance as a model system to quantify benzene dimer interactions using ^1^H‐NMR studies in various solvents. A comparison of the computationally most stable **1,6‐COT** conformers reveals that the **TT** and **PD** conformations (optimized at ωB97X‐D4/def2‐TZVPP [46, 47]) closely resemble the computed key experimental geometries of the gas‐phase benzene dimer [24]. Both the Ph‐Ph angles and distances also align well with those observed in computational gas‐phase studies of the benzene dimer [24, 25]. Additionally, the SAPT computations of the **TT** and **PD** conformations (Figure 3) are in very good agreement with the computed energies for the benzene dimer [24]. Therefore, the **1,4‐/1,6‐COT** balance is a suitable model to evaluate benzene dimer interaction energies in solution, which are otherwise challenging to quantify. The measured and computed **1,4/1,6‐**dibenzyl **COT** equilibrium shows a clear preference for folded (“more crowded”) **1,6‐COT**, irrespective of solvent. NCI plot and SAPT analyses (see Figures S19–S24 and Tables S18–S20) of the **TT** and **PD** structures support the conclusion that LD interactions are the primary stabilizing force governing the intramolecular interactions in **1,6‐COT** in solution. The derived experimental interaction energy in solution ranges from ∆*G*_(1,6/1,4)_  =  −0.23 kcal mol^−1^ (in benzene) up to ∆*G*_(1,6/1,4)_  =  −0.37 kcal mol^−1^ (in methanol) and is thereby considerably weaker than the computed gas phase energy of ∆*E*_int_ = −2.7 kcal mol^−1^.

## Conflicts of Interest

The authors declare no conflicts of interest.

## Supporting information



References from the Supporting Information are provided in the following listing as well: [62, 63, 64, 65, 66, 67, 68, 69, 70].
