# Supplementary material for: Assessing Benzene Dimer Interactions in Solution With a Molecular Balance
Source: Chemistry. 2026 May 18;32(33):e71126. doi: 10.1002/chem.71126 (PMC13532124; doi:10.1002/chem.71126)
Supplement: Supplementary file 1 [file CHEM-32-e71126-s001.pdf]

Electronic Supporting Information for

Assessing Benzene Dimer Interactions in Solution with a Molecular Balance

Marvin H. J. Domanski,[‡] Michael Fuhrmann,[‡] Nils F. Hacket, Heike Hausmann, Peter R. Schreiner*

[‡] Authors contributed equally to this work.

* Corresponding author

Institute of Organic Chemistry, Justus Liebig University, Heinrich-Buff-Ring 17, 35392 Giessen, Germany, E-mail: prs@uni-giessen.de

Content

1 Experimental Investigation 1

1.1 General Information 1

1.2 Van't Hoff plot 2

1.3 Equilibration time 4

1.4 Concentration Dependency 5

1.5 Equilibrium in of 1,4/1,6-COT in different solvents. 6

1.6 Proton assignment of 1,4/1,6-COT 18

2 Computational Evaluation 19

2.1 Equilibrium energies in solution 19

2.2 Thermodynamic data and coordinates of TT-1,6-COT and PD-1,6-COT. 22

2.3 Noncovalent interaction plots 28

2.4 SAPT Analysis 29

2.5 Local Energy Decomposition (LED) 32

2.6 Computed ^{1}H and ^{13}C NMR Spectrum 34

3 Synthetic Details 35

3.1 General Information 35

3.2 Synthetic procedures 35

3.3 NMR Spectra 37

4 References 39

1 Experimental Investigation

1.1 General Information

NMR spectra were obtained using either Bruker Avance II 400 MHz, Bruker Avance III HD 400 MHz, Bruker Avance III HD 600 MHz, or Bruker Avance Neo 700 MHz spectrometers. High resolution mass spectra were obtained utilizing a Bruker microTOF mass spectrometer.

Evaluation of NMR Spectra

K_{eq} for the equilibrium of **1,4/1,6-COT** in a respective solvent, was determined by integration of either methylene protons. A weak splitting of the benzylic signal can be observed at low temperatures, at temperatures of 40 °C and above the splitting vanished. Prior to each measurement, unless otherwise noted, the sample was equilibrated for at least 12 h at 40 °C. ^{1}H-NMR experiments were carried out with a Bruker Avance III HD 600 MHz spectrometer. ΔG_{eq} was then calculated according to the following equation:

$$\Delta_r G^{313.15\,K} = -\ln(K_{eq})\,RT \tag{1}$$

The total differential was utilized for calculating error bars assuming an integration error of 5% and ΔT = 0.15 K.

$$\Delta\Delta_r G^{313.15\,K} = \left|-\frac{RT}{K_{eq}}\right| \Delta K_{eq} + \left|-\ln(K_{eq})\; R\right| \Delta T \tag{2}$$

Examples for signal assignment/integration are shown below:

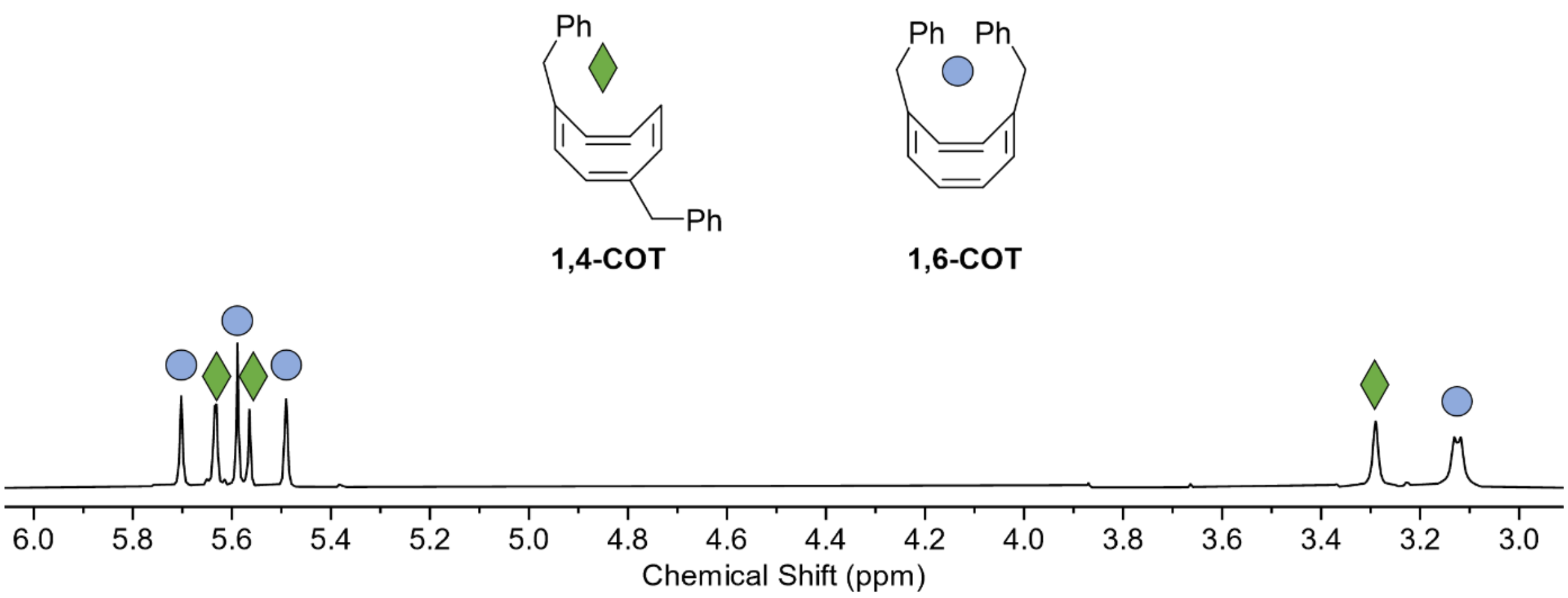

Figure S1: Assignment of benzylic signals to **1,4/1,6-COT**.

1.2 Van’t Hoff plot

The Van't Hoff analysis has been performed in a temperature range of 308 – 343 K according to equation 7 in it is modified form and is depicted in Figure S2. The recorded Van’t Hoff data reveal an enthalpic contribution of ΔH = −0.52 ± 0.03 kcal mol^{-1} with an entropy contribution of ΔS = −0.722±0.081 cal mol^{-1} K^{-1}. The equilibrium is *exergonic* in favor of the 1,6-isomer. Additionally, we provided the original Van’t Hoff diagram in Figure S3.

$$\Delta_r \boldsymbol{G}^{\theta} = \boldsymbol{\Delta_r H^{\theta}} - \boldsymbol{T\Delta_r S^{\theta}} \tag{3}$$

$$\boldsymbol{K_{eq}} = \boldsymbol{e}^{-\frac{\Delta_r \boldsymbol{G}^{\theta}}{\boldsymbol{RT}}} \tag{4}$$

$$\boldsymbol{K_{eq}} = \boldsymbol{e}^{-\frac{\boldsymbol{\Delta_r H^{\theta}} - \boldsymbol{T\Delta_r S^{\theta}}}{\boldsymbol{RT}}} \tag{5}$$

$$\ln \boldsymbol{K_{eq}} = -\frac{\boldsymbol{\Delta_r H^{\theta}} - \boldsymbol{T\Delta_r S^{\theta}}}{\boldsymbol{RT}} \tag{6}$$

$$-\boldsymbol{R}\ln \boldsymbol{K_{eq}} = \boldsymbol{\Delta_r H^{\theta}}\frac{\mathbf{1000}}{\boldsymbol{T}}[\boldsymbol{kcal\,mol^{-1}}] - \Delta_r \boldsymbol{S}^{\theta}[\boldsymbol{cal\,mol^{-1}}] \tag{7}$$

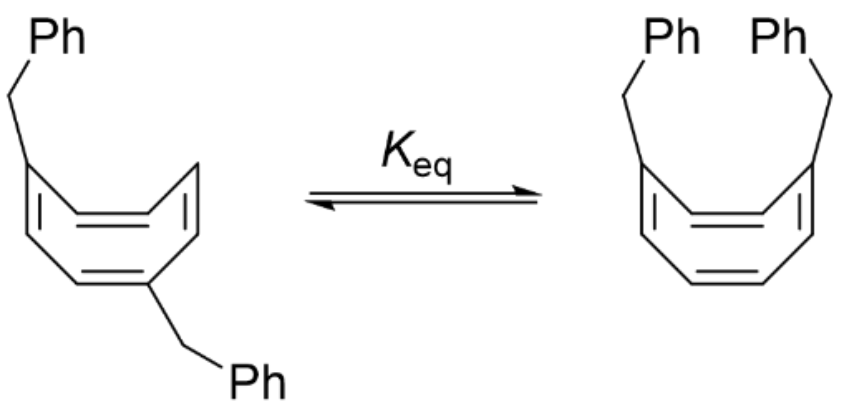

1,4-COT **1,6-COT**

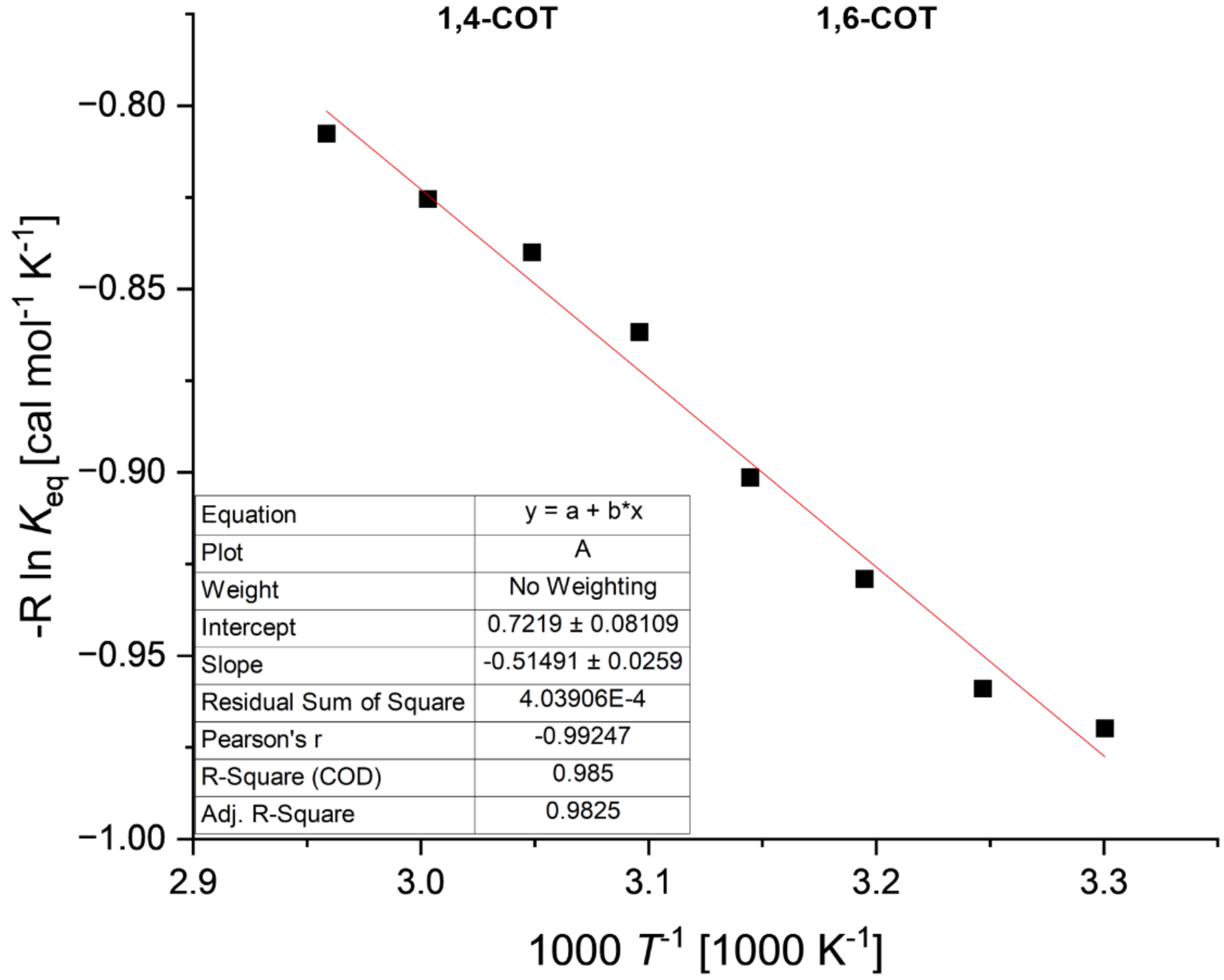

Figure S2: Modified Van’t Hoff analysis of the **1,4/1,6-COT** equilibrium in DCE-d_4 following equation 7.

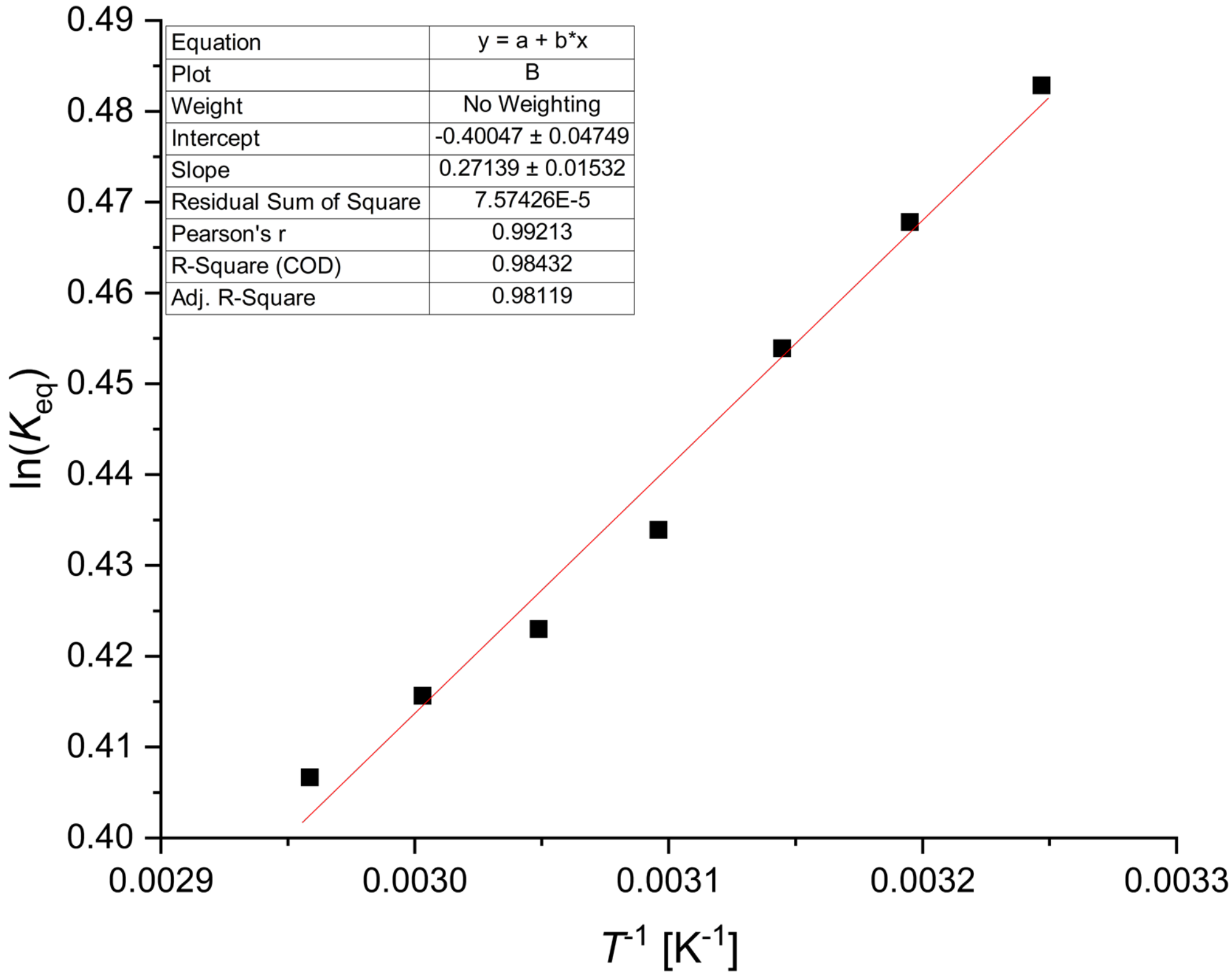

Figure S3: Original van't Hoff analysis of the **1,4/1,6-COT** equilibrium in DCE-d_4.

1.3 Equilibration time

To determine a suitable equilibrium temperature for the isomeric **1,4/1,6-COT** mixture, and investigate the change of K_{eq} over time, we tempered a probe in DCE-d_4 to 70 °C, cooled to 40 °C and measured the 1,4/1,6-ratio every 5 min. The equilibration constant K_{eq} settled immediately and remained unaffected over time. Indicating that the equilibrium settled fast.

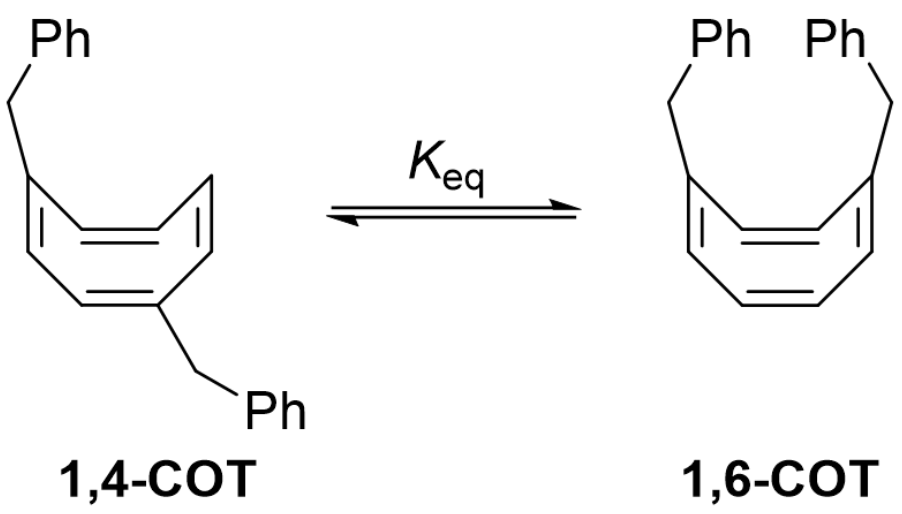

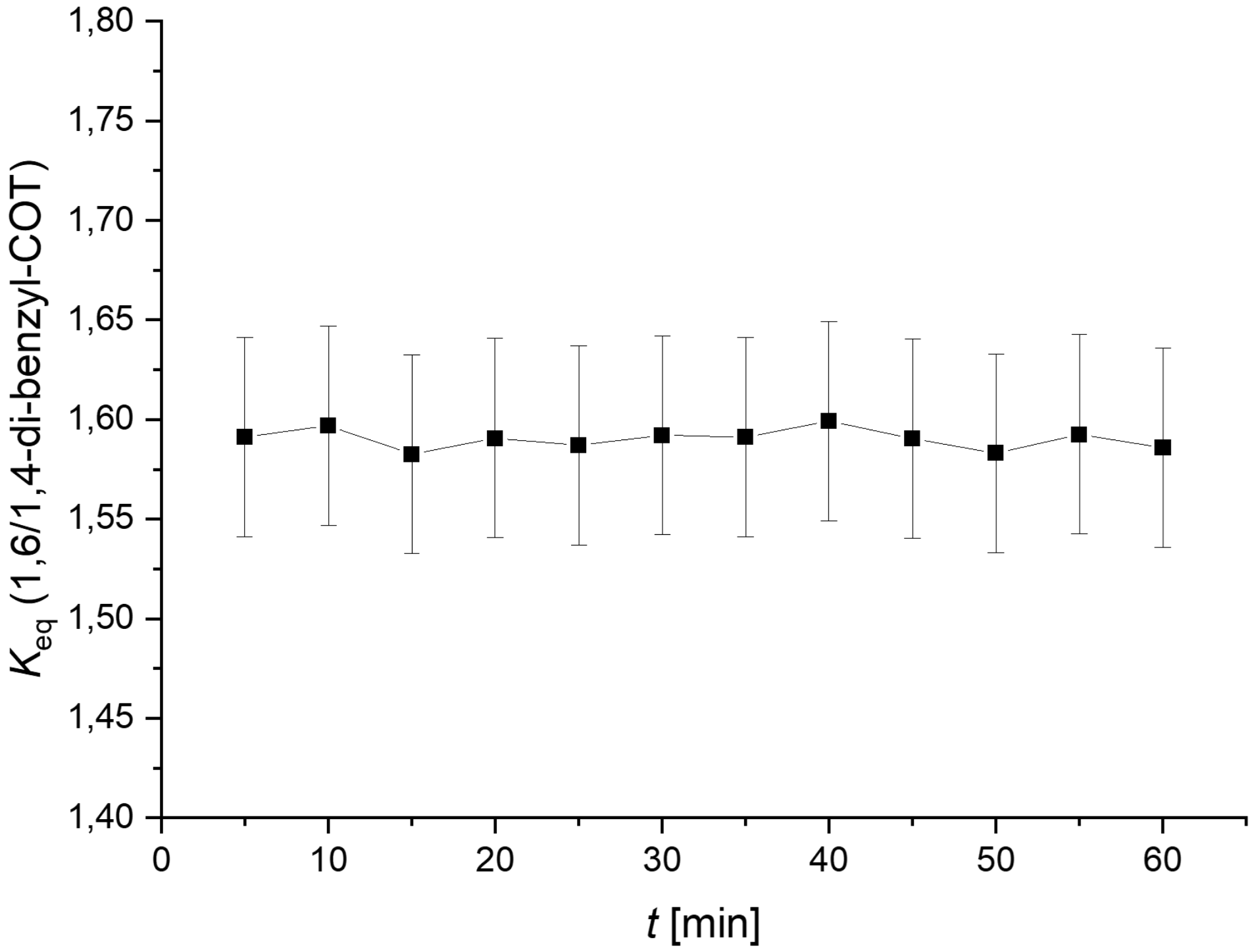

Figure S4: Equilibrium study of the **1,4/1,6-COT** interconversion. NMR measurements were performed in DCE-d_4 at 40 °C.

1.4 Concentration Dependency

To investigate the influence of different **1,4/1,6-COT** concentrations on the equilibrium constant K_{eq} we measured ^{1}H-NMRs of samples with 6, 12, 30, and 60 mmol in 0.6 mL $CDCl_3$ at 40 °C. The equilibrium constant remained constant over different concentrations.

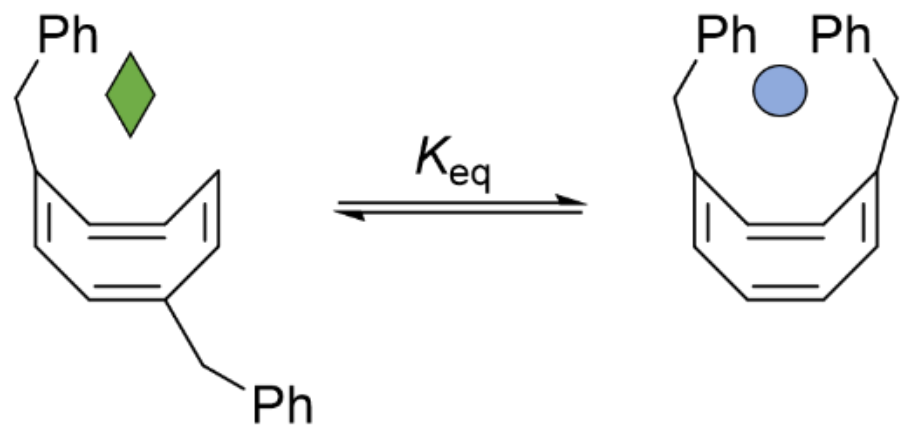

1,4-COT **1,6-COT**

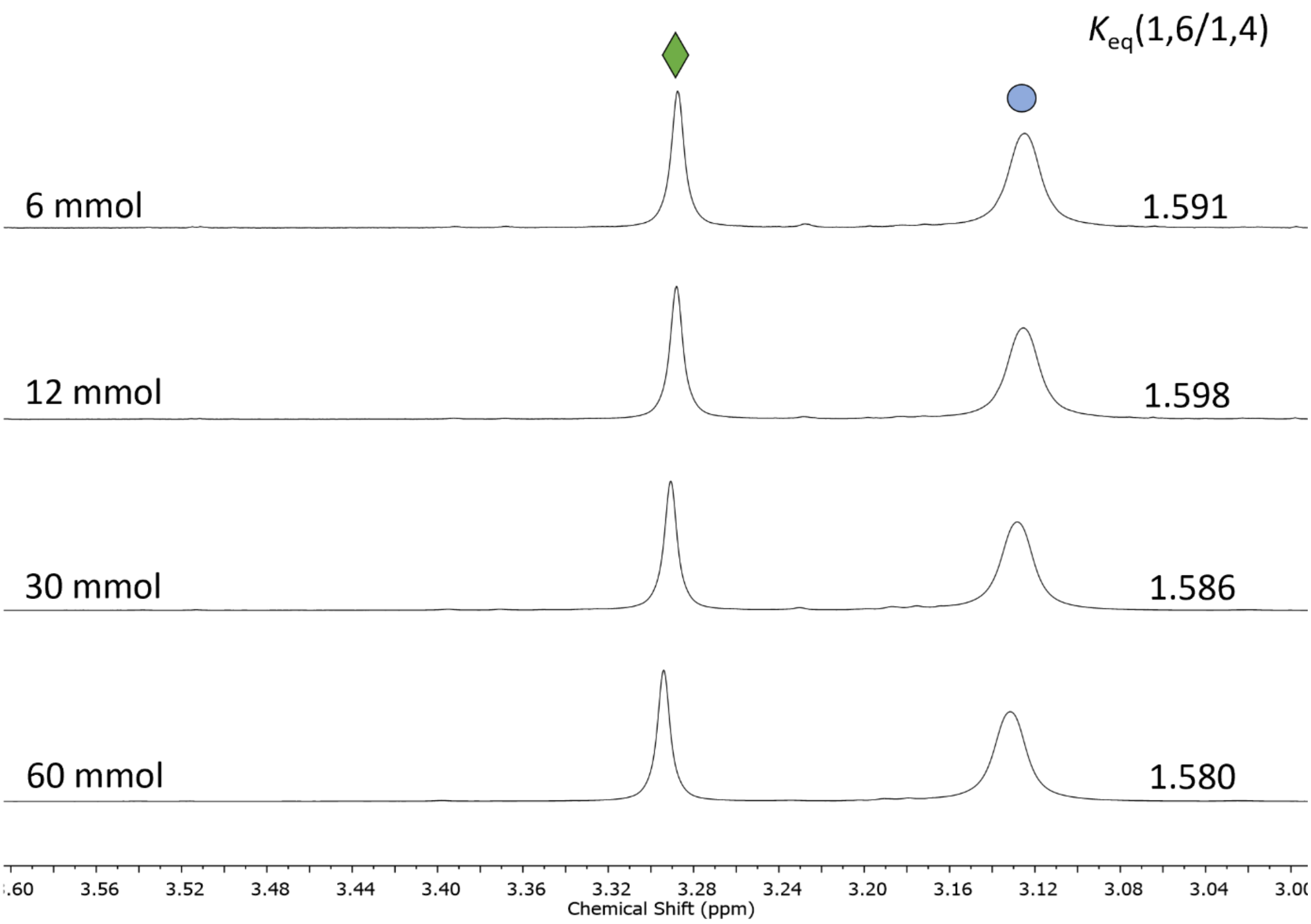

Figure S5: NMR measurements of different **1,4/1,6-COT** concentrations at 40 °C in $CDCl_3$.

1.5 Equilibrium in of 1,4/1,6-COT in different solvents.

Methanol-d_4

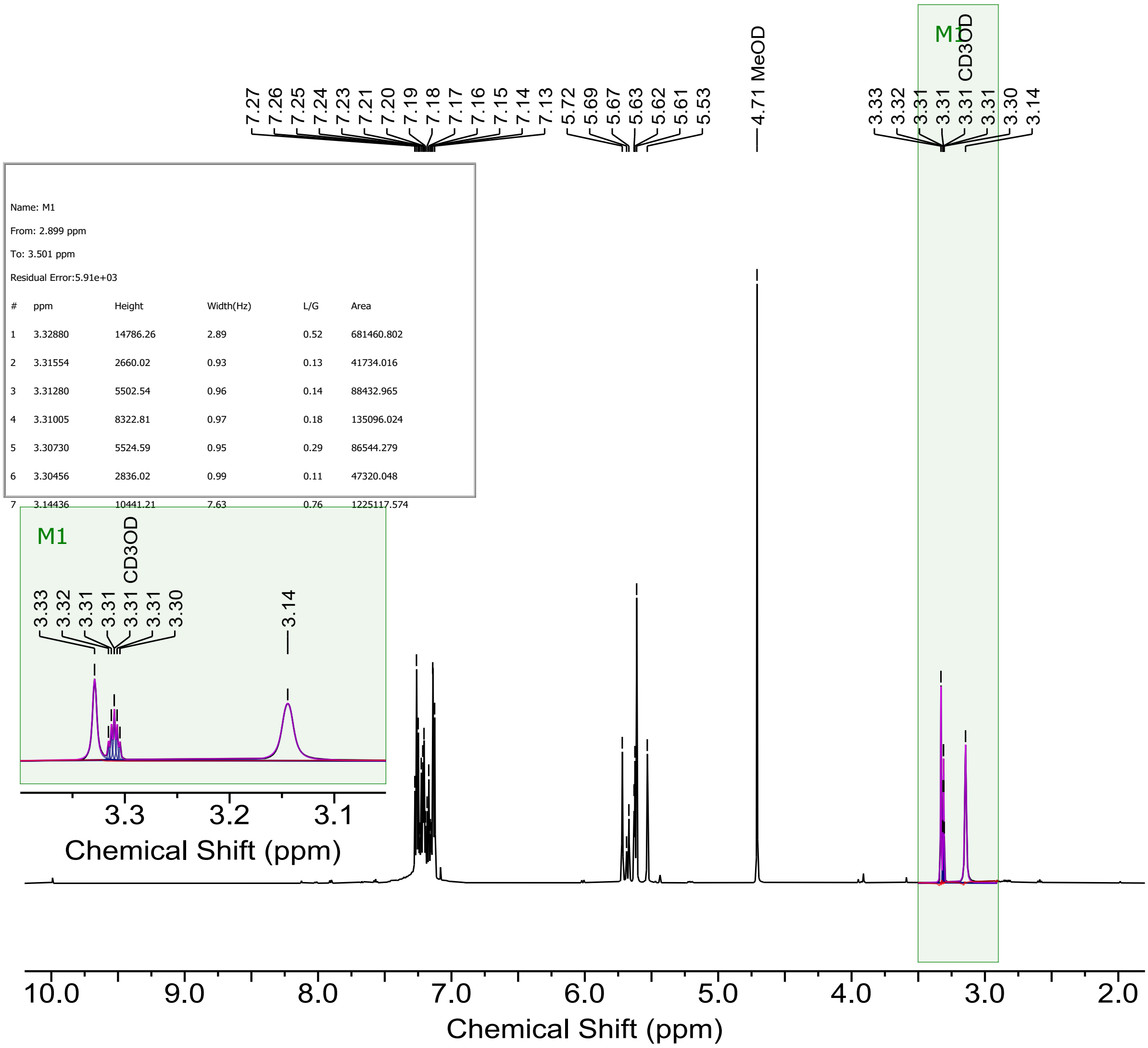

Figure S6: 600 MHz ^{1}H-NMR of **1,4/1,6-COT** in methanol-d_4 at 313.15 K.

Table S1: Equilibrium constants K_{eq} and Gibbs free energies (in kcal mol^{-1}) of **1,4/1,6-COT** in methanol-d_4 obtained from the corresponding NMR integration.

solvent	K_{eq}	ΔK_{eq}	ΔG_{eq}	$\Delta\Delta G_{eq}$
methanol-d_4	1.80	±0.09	−0.36	±0.03

n-Hexane-d14

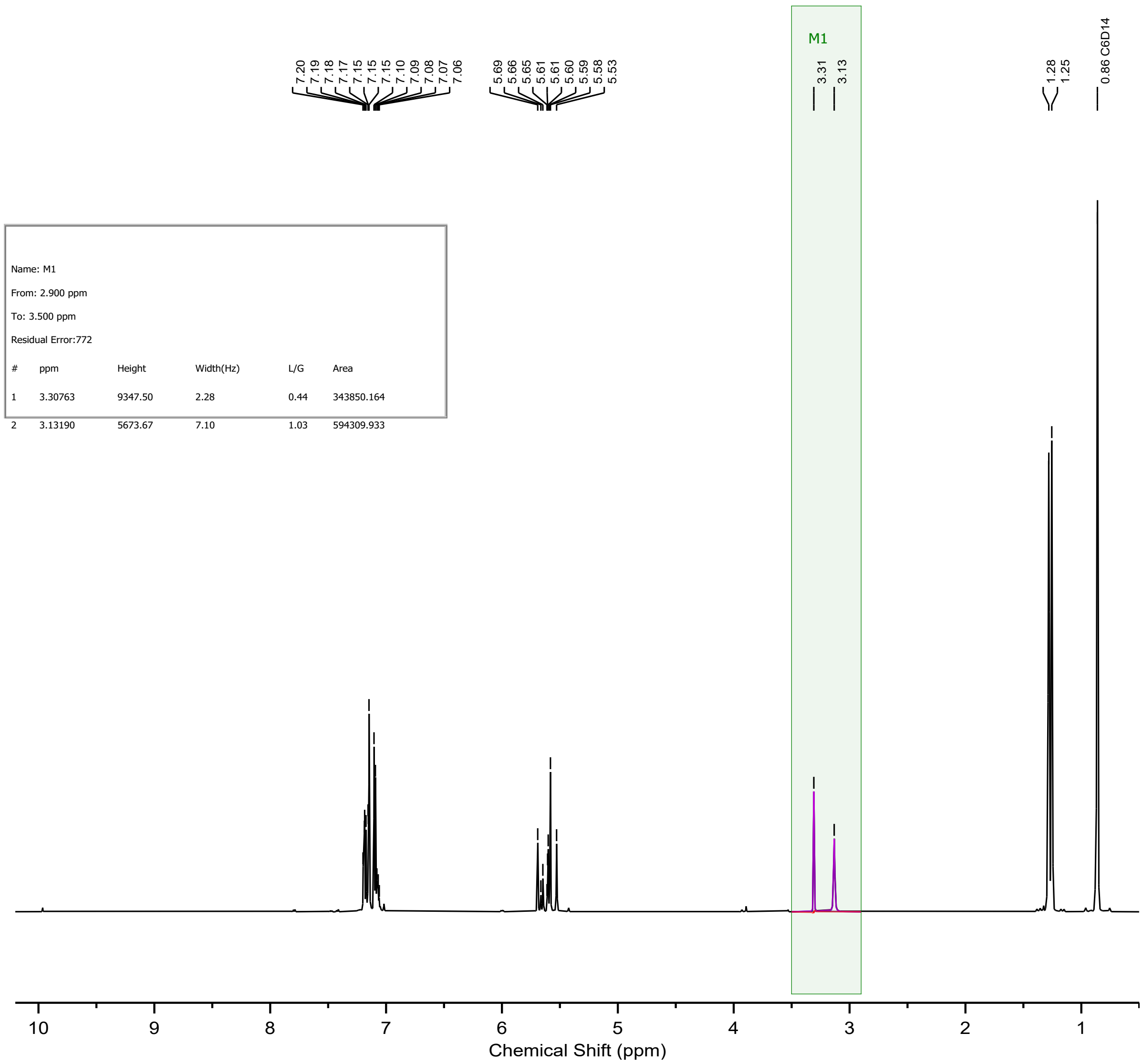

Figure S7: 600 MHz ^{1}H-NMR of **1,4/1,6-COT** in *n*-hexane-*d*14 at 313.15 K.

Table S2: Equilibrium constants K_{eq} and Gibbs free energies (in kcal mol^{-1}) of **1,4/1,6-COT** in *n*-hexane-*d*14 obtained from the corresponding NMR integration.

solvent	K_{eq}	ΔK_{eq}	ΔG_{eq}	$\Delta\Delta G_{eq}$
n-hexane-*d*14	1.73	±0.09	−0.34	±0.03

Acetonitrile-d_3

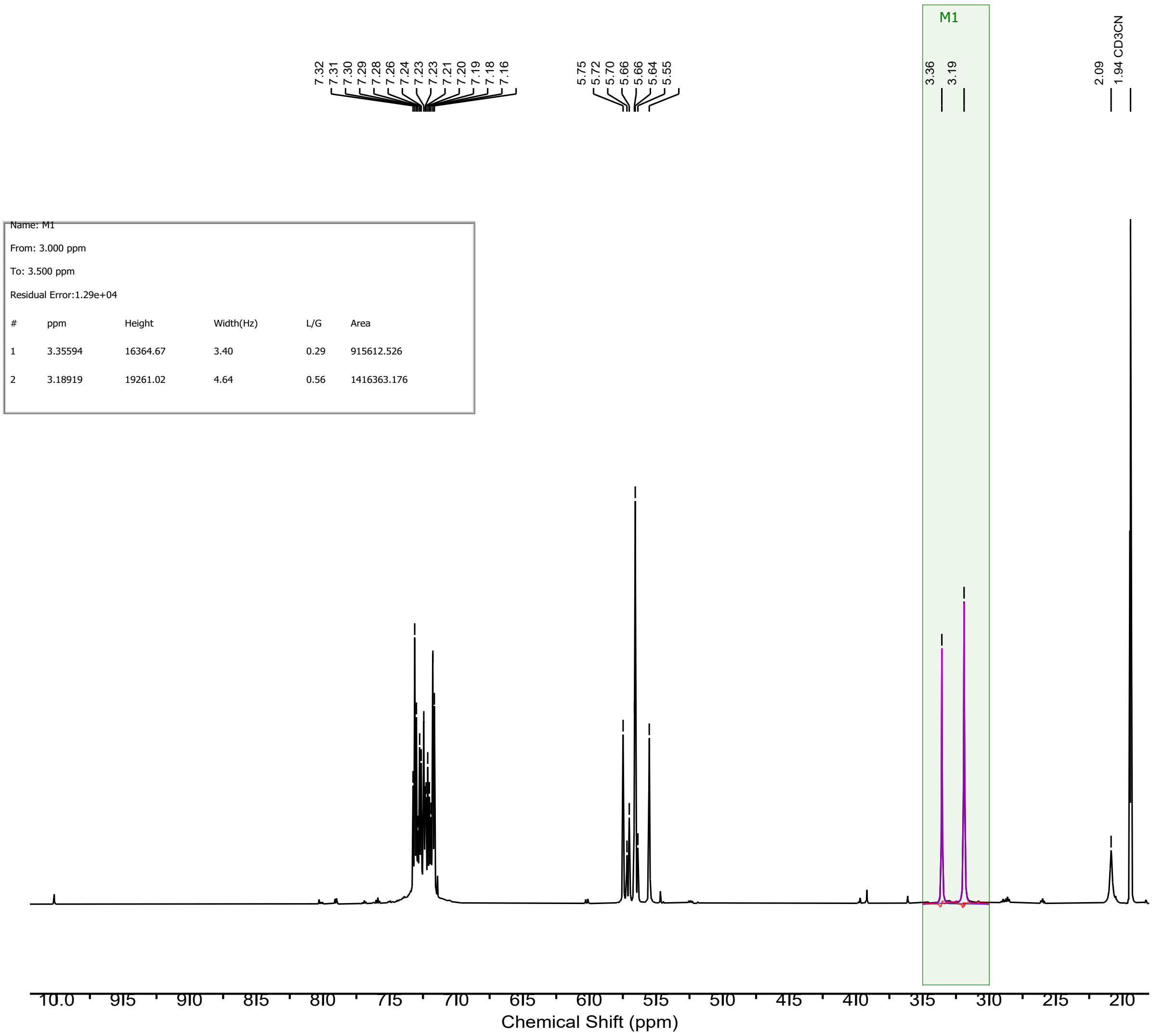

Figure S8: 600 MHz ^{1}H-NMR of **1,4/1,6-COT** in acetonitrile-d_3 at 313.15 K.

Table S3: Equilibrium constants K_{eq} and Gibbs free energies (in kcal mol^{-1}) of **1,4/1,6-COT** in acetonitrile-d_3 obtained from the corresponding NMR integration.

solvent	K_{eq}	ΔK_{eq}	ΔG_{eq}	$\Delta\Delta G_{eq}$
acetonitrile-d_3	1.55	±0.08	−0.27	±0.03

Acetone-d_6

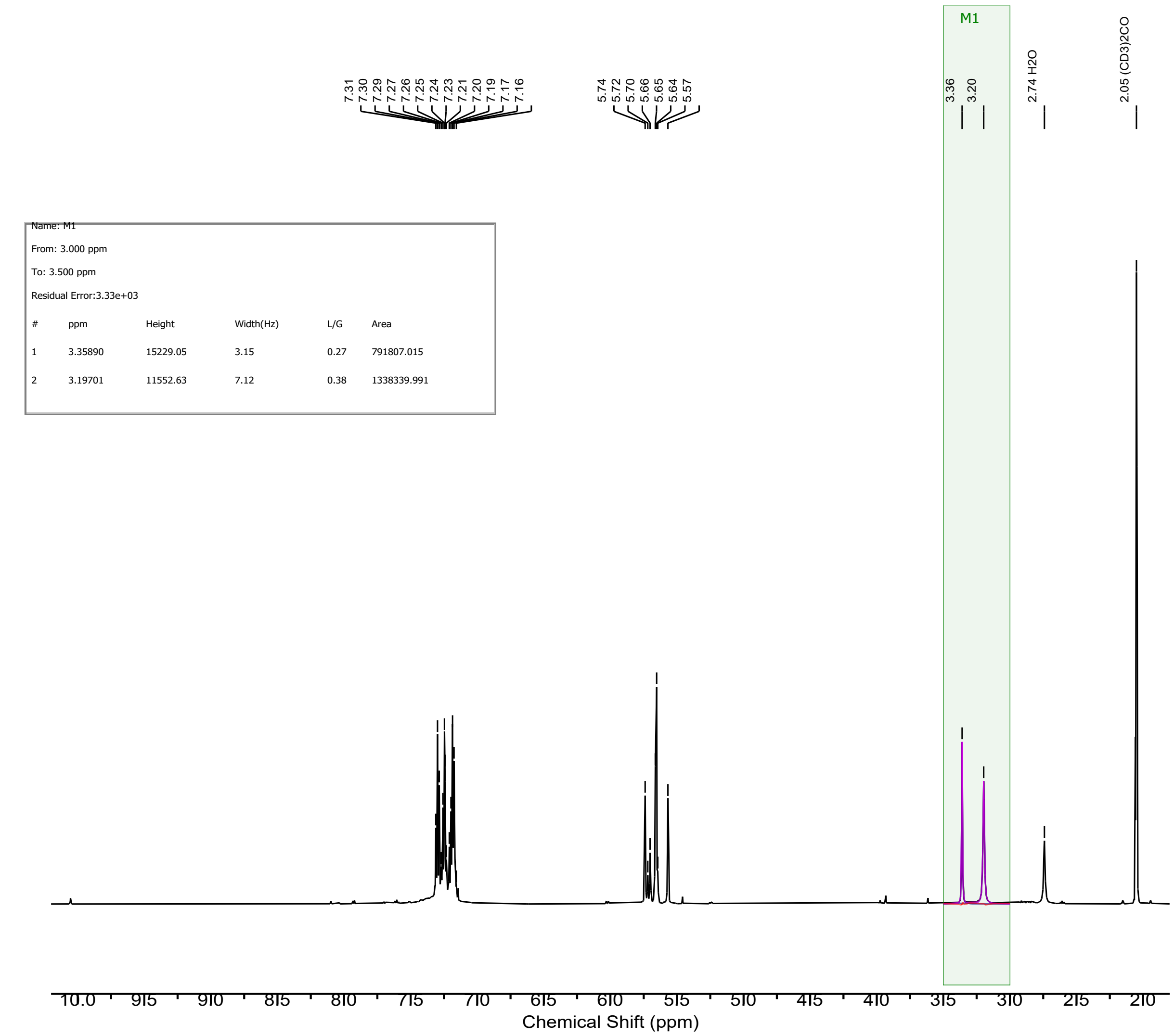

Figure S9: 600 MHz ^{1}H-NMR of **1,4/1,6-COT** in acetone-d_6 at 313.15 K.

Table S4: Equilibrium constants K_{eq} and Gibbs free energies (in kcal mol^{-1}) of **1,4/1,6-COT** in acetone-d_6 obtained from the corresponding NMR integration.

solvent	K_{eq}	ΔK_{eq}	ΔG_{eq}	$\Delta\Delta G_{eq}$
acetone-d_6	1.69	±0.08	−0.32	±0.03

Cyclohexane-d_{12}

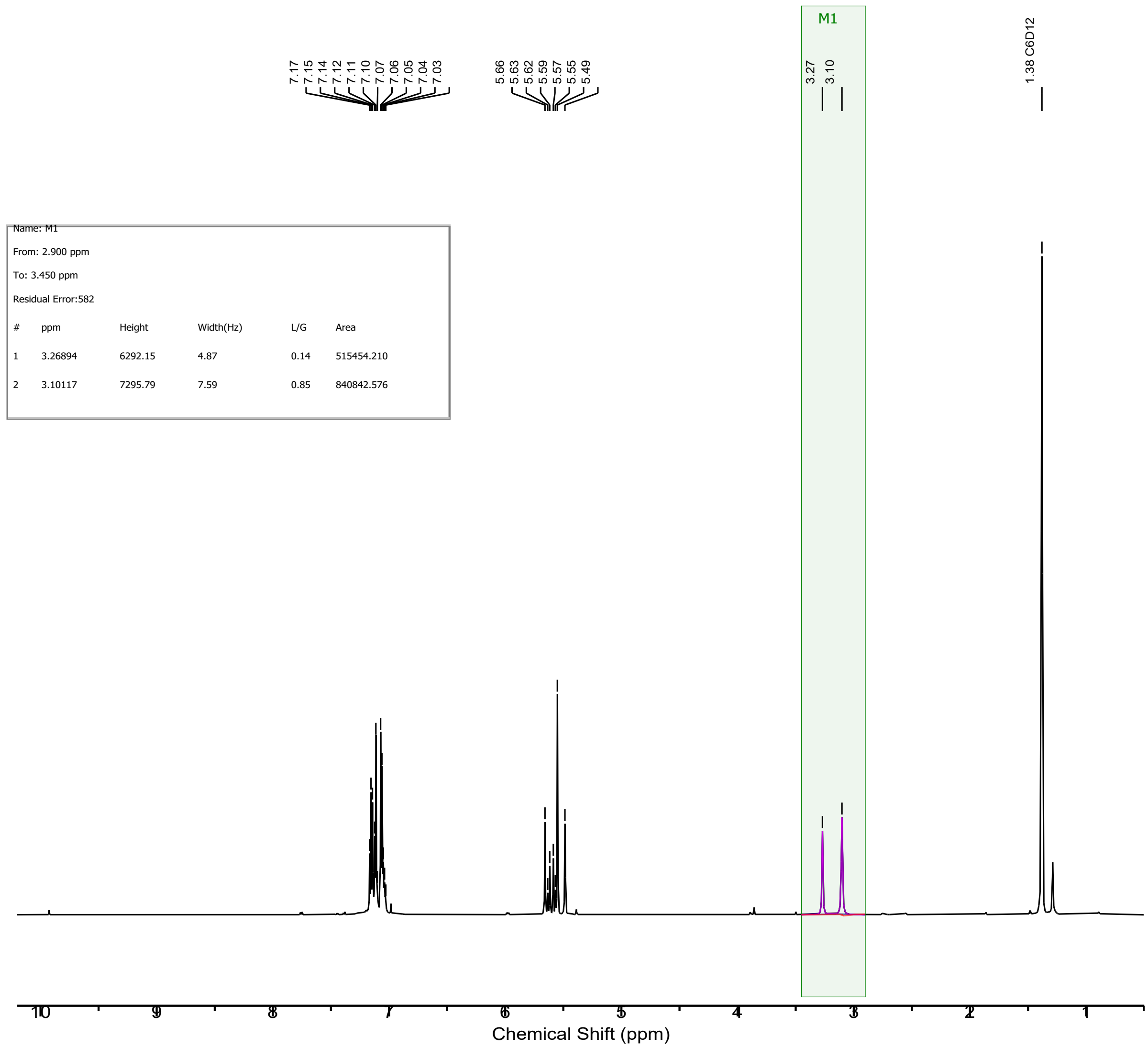

Figure S10: 600 MHz ^{1}H-NMR of **1,4/1,6-COT** in cyclohexane-d_{12} at 313.15 K.

Table S5: Equilibrium constants K_{eq} and Gibbs free energies (in kcal mol^{-1}) of **1,4/1,6-COT** in cyclohexane-d_{12} obtained from the corresponding NMR integration.

solvent	K_{eq}	ΔK_{eq}	ΔG_{eq}	$\Delta\Delta G_{eq}$
cyclohexane-d_{12}	1.63	±0.08	−0.30	±0.03

Tetrahydrofuran-d_8

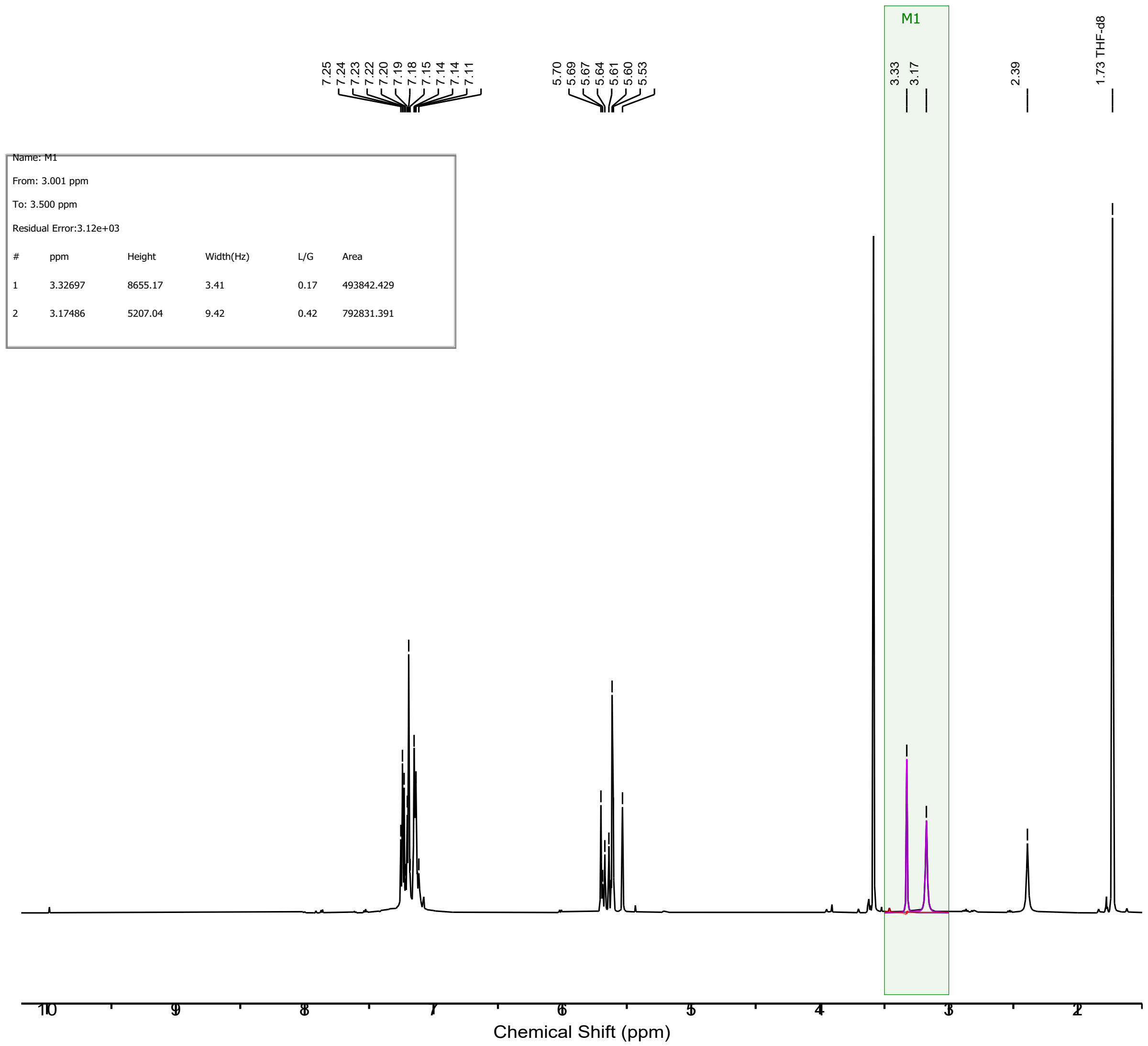

Figure S11: 600 MHz ^{1}H-NMR of **1,4/1,6-COT** in tetrahydrofuran-d_8 at 313.15 K.

Table S6: Equilibrium constants K_{eq} and Gibbs free energies (in kcal mol^{-1}) of **1,4/1,6-COT** in tetrahydrofuran-d_8 obtained from the corresponding NMR integration.

solvent	K_{eq}	ΔK_{eq}	ΔG_{eq}	$\Delta\Delta G_{eq}$
tetrahydrofuran-d_8	1.60	±0.08	−0.29	±0.03

1,2-Dichloroethane-d_4

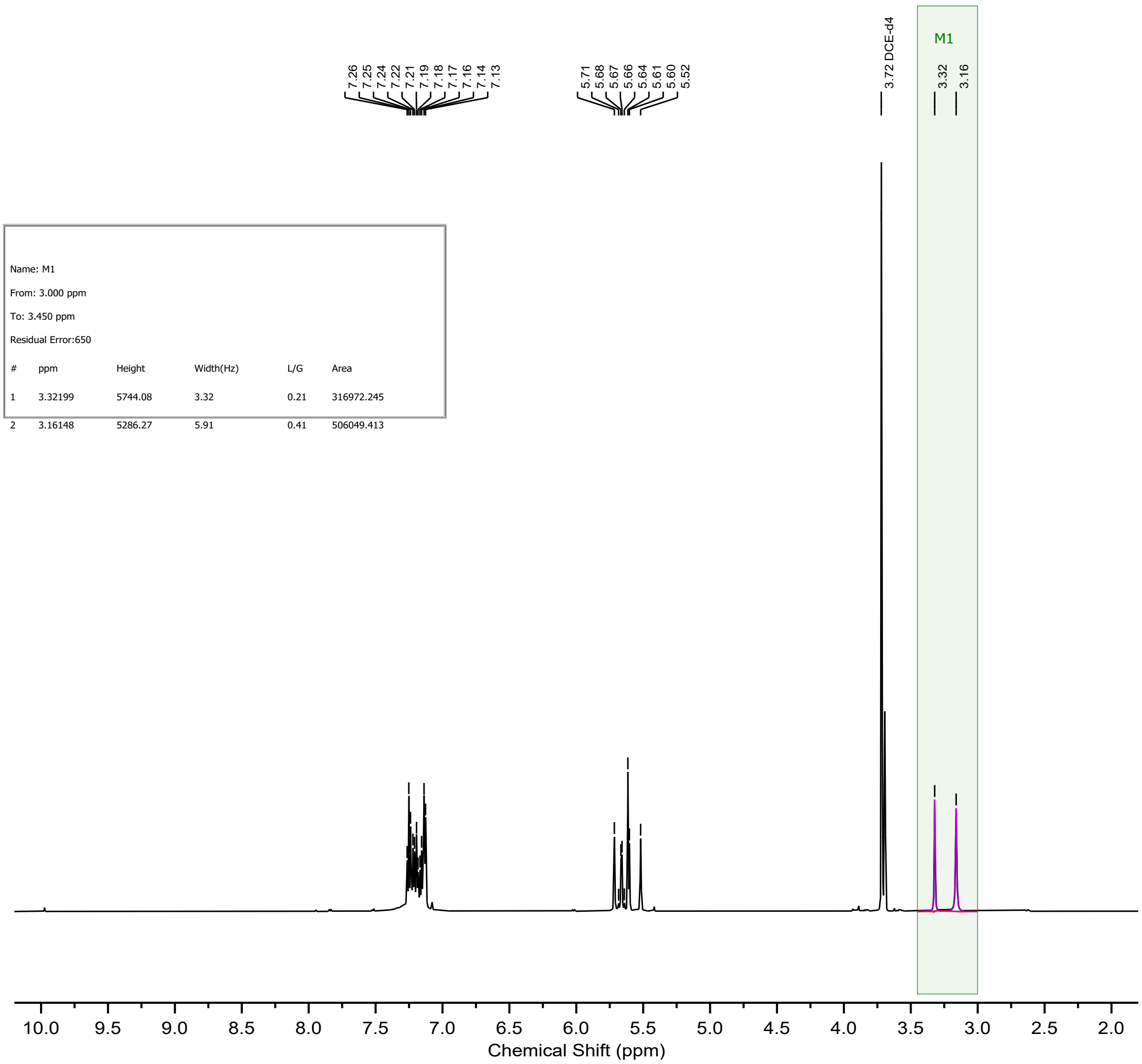

Figure S12: 600 MHz ^{1}H-NMR of **1,4/1,6-COT** in 1,2-dichloroethane-d_4 at 313.15 K.

Table S7: Equilibrium constants K_{eq} and Gibbs free energies (in kcal mol^{-1}) of **1,4/1,6-COT** in 1,2-dichloroethane-d_4 obtained from the corresponding NMR integration.

solvent	K_{eq}	ΔK_{eq}	ΔG_{eq}	$\Delta\Delta G_{eq}$
1,2-dichloroethane-d_4	1.60	±0.08	−0.29	±0.03

Toluene-d_8

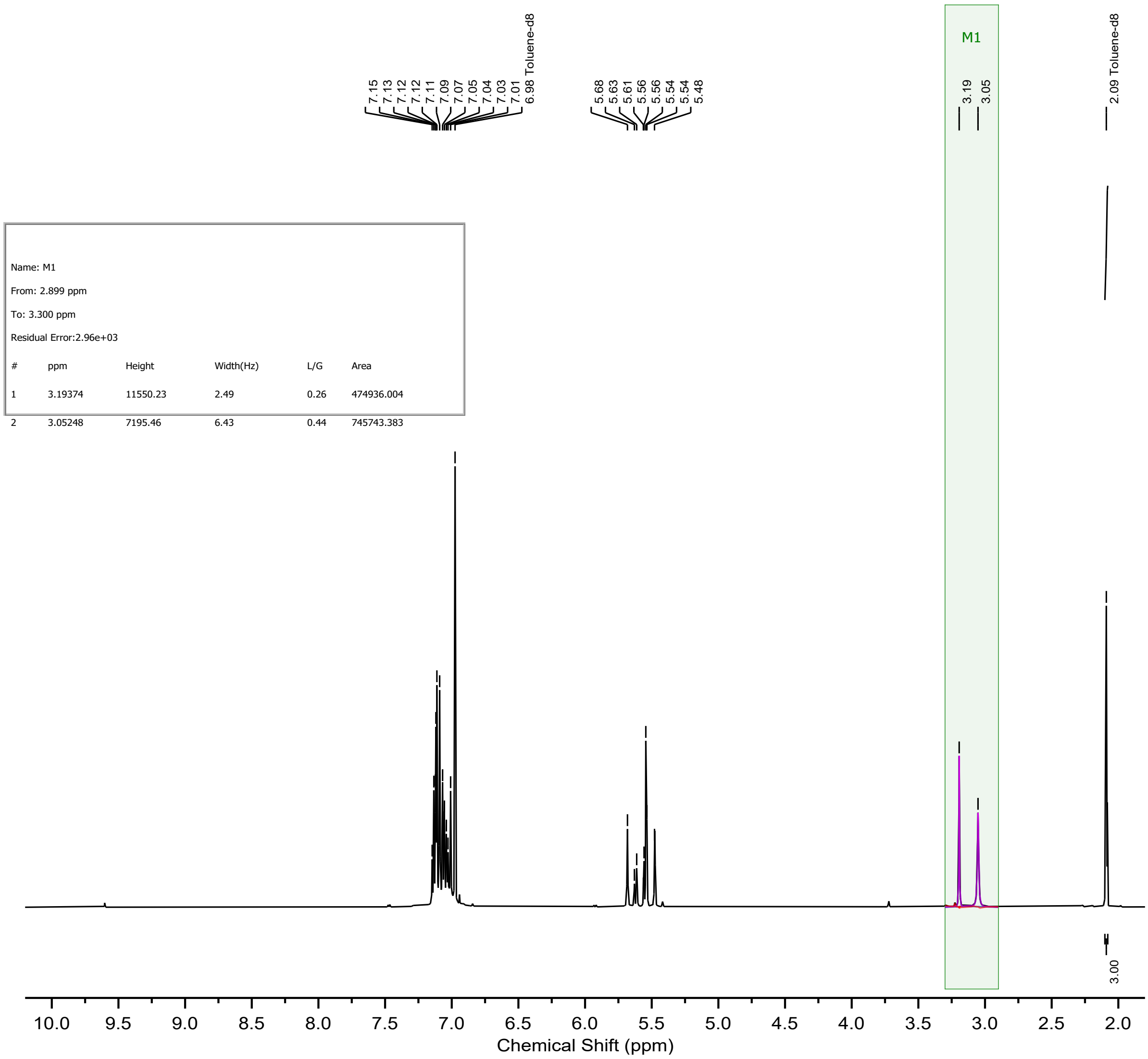

Figure S13: 600 MHz ^{1}H-NMR of **1,4/1,6-COT** in toluene-d_8 at 313.15 K.

Table S8: Equilibrium constants K_{eq} and Gibbs free energies (in kcal mol^{-1}) of **1,4/1,6-COT** in toluene-d_8 obtained from the corresponding NMR integration.

solvent	K_{eq}	ΔK_{eq}	ΔG_{eq}	$\Delta\Delta G_{eq}$
toluene-d_8	1.57	±0.08	−0.28	±0.03

Chloroform-d_1

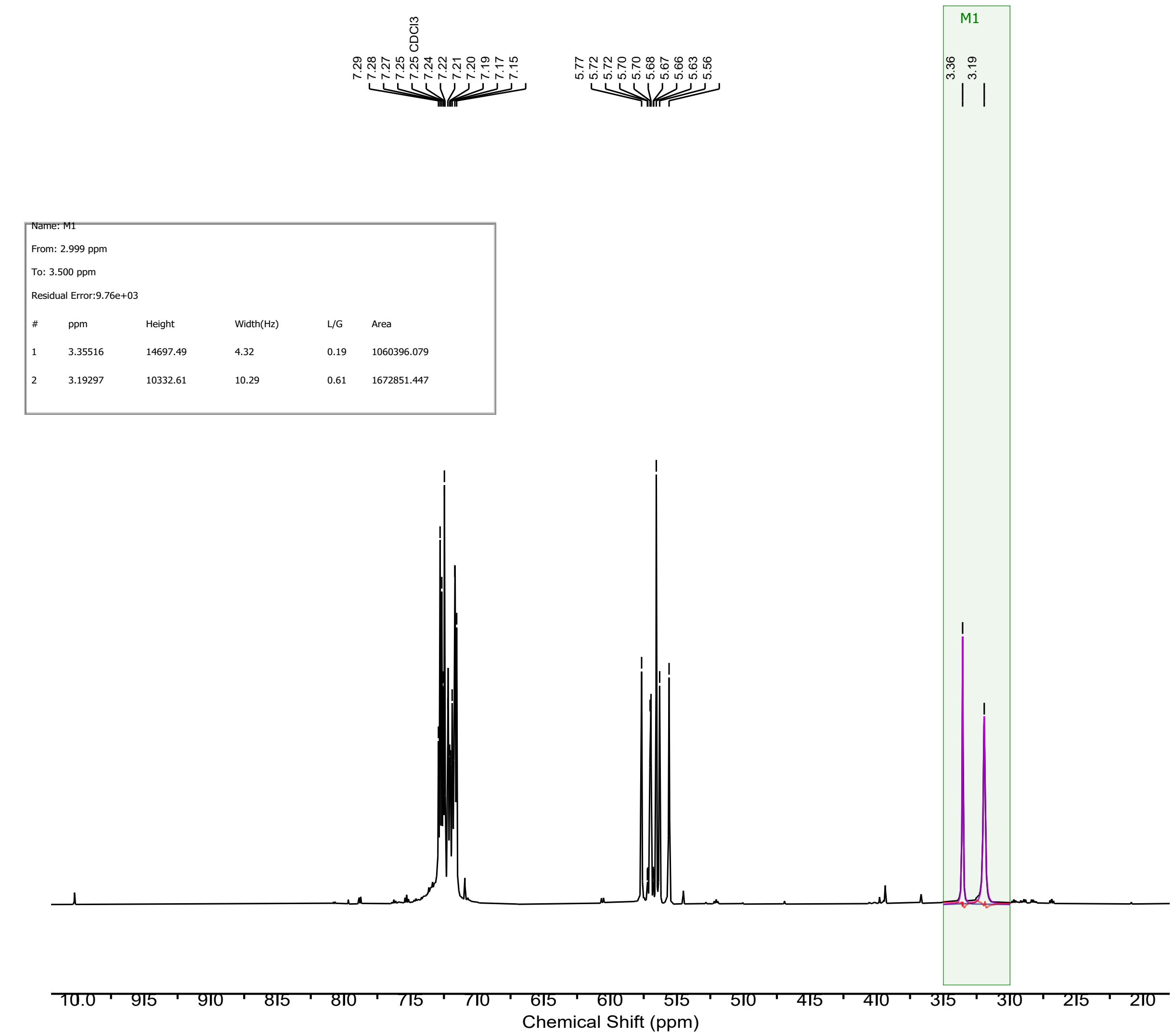

Figure S14: 600 MHz ^{1}H-NMR of **1,4/1,6-COT** in chloroform-d1 at 313.15 K.

Table S9: Equilibrium constants K_{eq} and Gibbs free energies (in kcal mol^{-1}) of **1,4/1,6-COT** in chloroform-d_1 obtained from the corresponding NMR integration.

solvent	K_{eq}	ΔK_{eq}	ΔG_{eq}	$\Delta\Delta G_{eq}$
chloroform-d_1	1.58	±0.08	−0.28	±0.03

Benzene-d_6

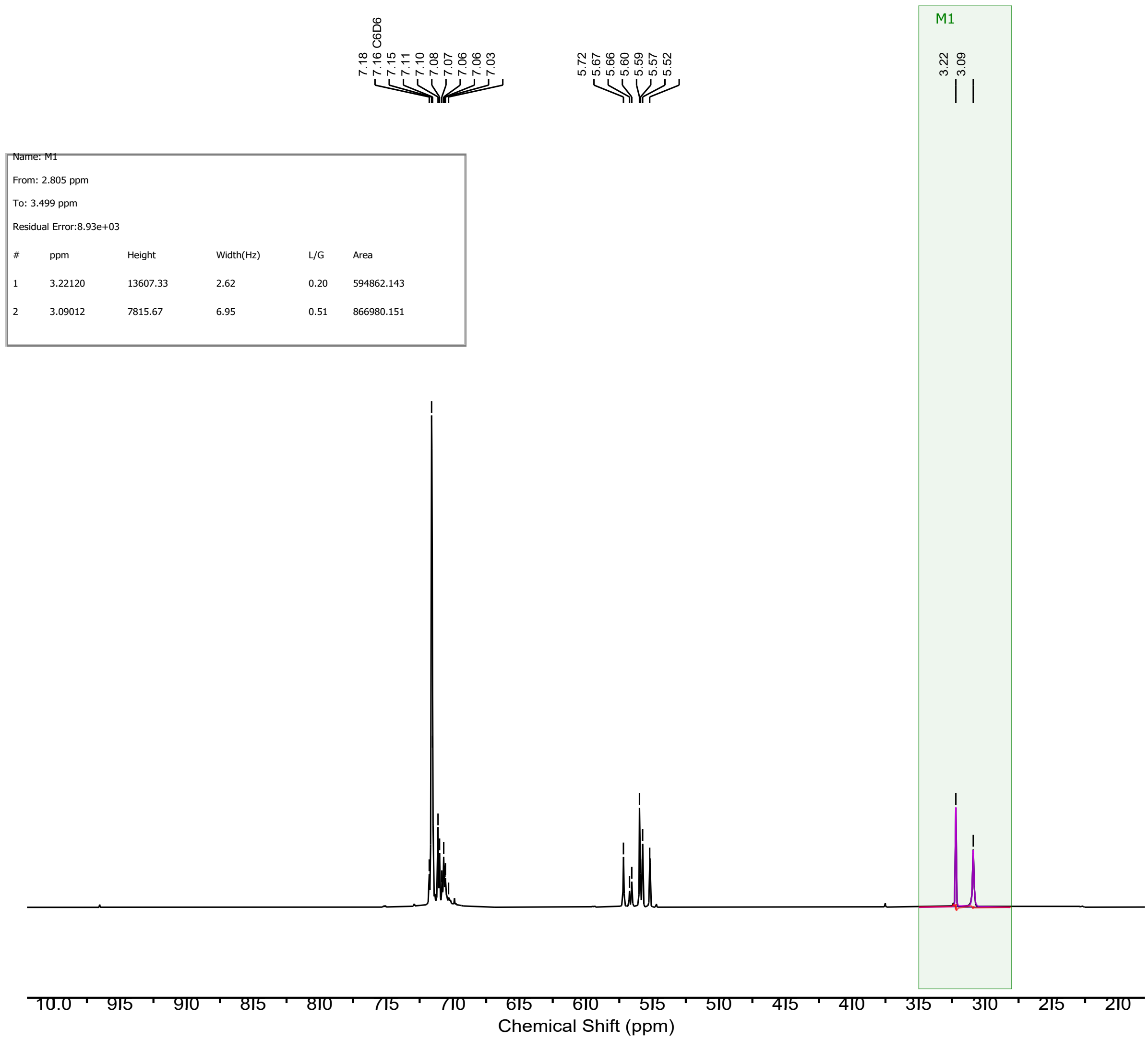

Figure S15: 600 MHz ^{1}H-NMR of **1,4/1,6-COT** in benzene-d$_6$ at 313.15 K.

Table S10: Equilibrium constants K_{eq} and Gibbs free energies (in kcal mol^{-1}) of **1,4/1,6-COT** in benzene-d_6 obtained from the corresponding NMR integration.

solvent	K_{eq}	ΔK_{eq}	ΔG_{eq}	$\Delta\Delta G_{eq}$
benzene-d$_6$	1.46	±0.07	−0.23	±0.03

Dimethylsulfoxide-d_6

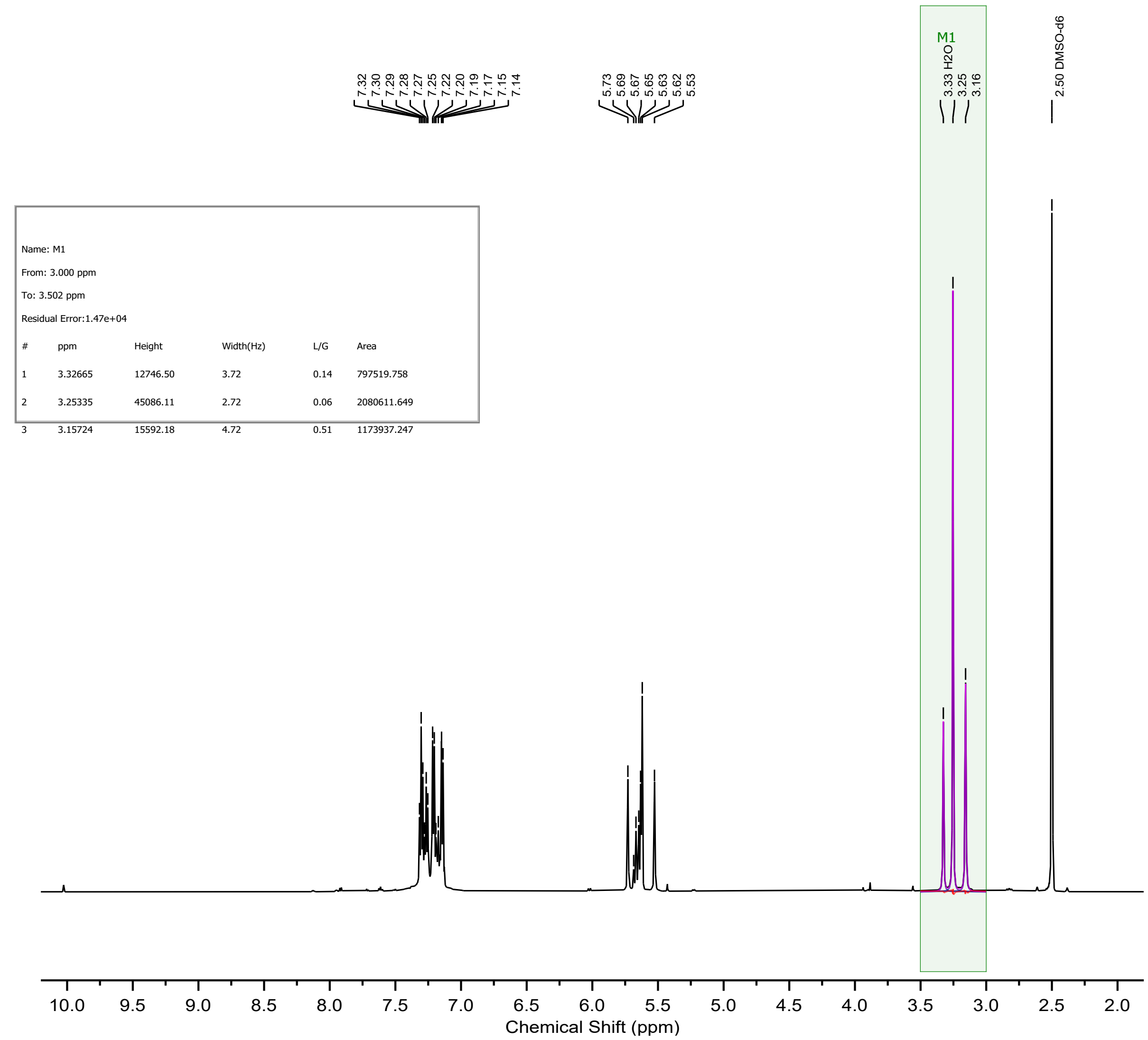

Figure S16: 600 MHz ^{1}H-NMR of **1,4/1,6-COT** in dimethylsulfoxide-d_6 at 313.15 K.

Table S11: Equilibrium constants K_{eq} and Gibbs free energies (in kcal mol^{-1}) of **1,4/1,6-COT** in dimethylsulfoxide-d_6 obtained from the corresponding NMR integration.

solvent	K_{eq}	ΔK_{eq}	ΔG_{eq}	$\Delta\Delta G_{eq}$
dimethylsulfoxide-d_6	1.77	±0.09	−0.36	±0.03

Carbondisulfide (locked on benzene-d_6)

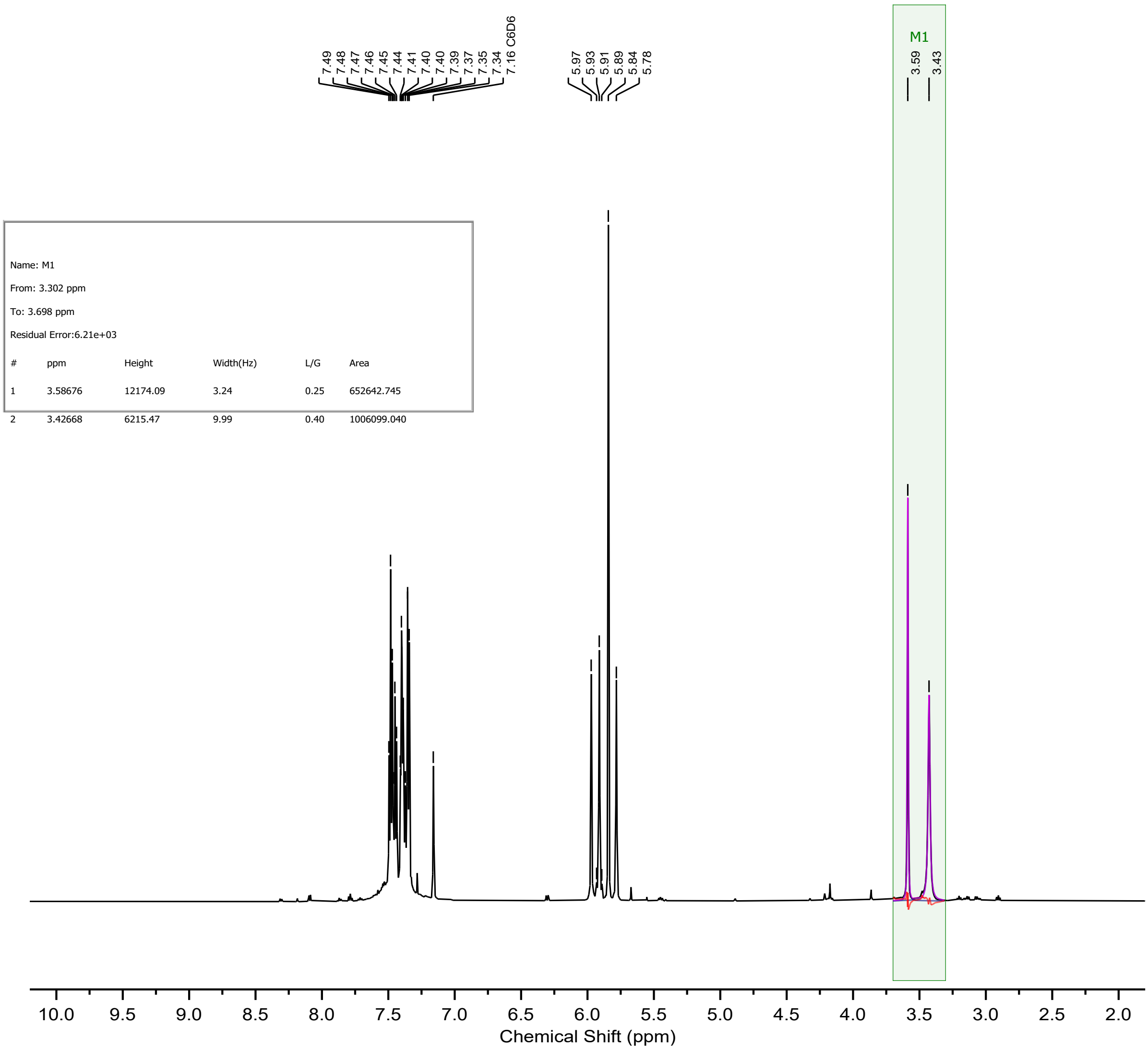

Figure S17: 600 MHz ^{1}H-NMR of **1,4/1,6-COT** in carbondisulfide (locked on benzene-d_6) at 313.15 K.

Table S12: Equilibrium constants Keq and Gibbs free energies (in kcal mol^{-1}) of **1,4/1,6-COT** in carbondisulfide (locked on benzene-d_6) obtained from the corresponding NMR integration.

solvent	K_{eq}	ΔK_{eq}	ΔG_{eq}	$\Delta\Delta G_{eq}$
carbondisulfide	1.54	±0.08	−0.27	±0.03

1.6 Proton assignment of 1,4/1,6-COT

The corresponding protons of **1,4/1,6-COT** have been assigned according to Figure S18. The olefinic 1,6-COT backbone at 5.70 and 5.49 ppm correlates with the corresponding benzylic protons at 3.12 ppm. The signal at 5.59 ppm does only show weak interaction with the benzylic protons at 3.12 ppm, however, it shows the same intensity as the other olefinic 1,6-COT signals. On the other hand, the 1,4-COT protons at 5.63 and 5.56 ppm correlate with the benzylic protons at 3.29 ppm. Assignment of the aromatic signals was not possible due to overlapping signals.

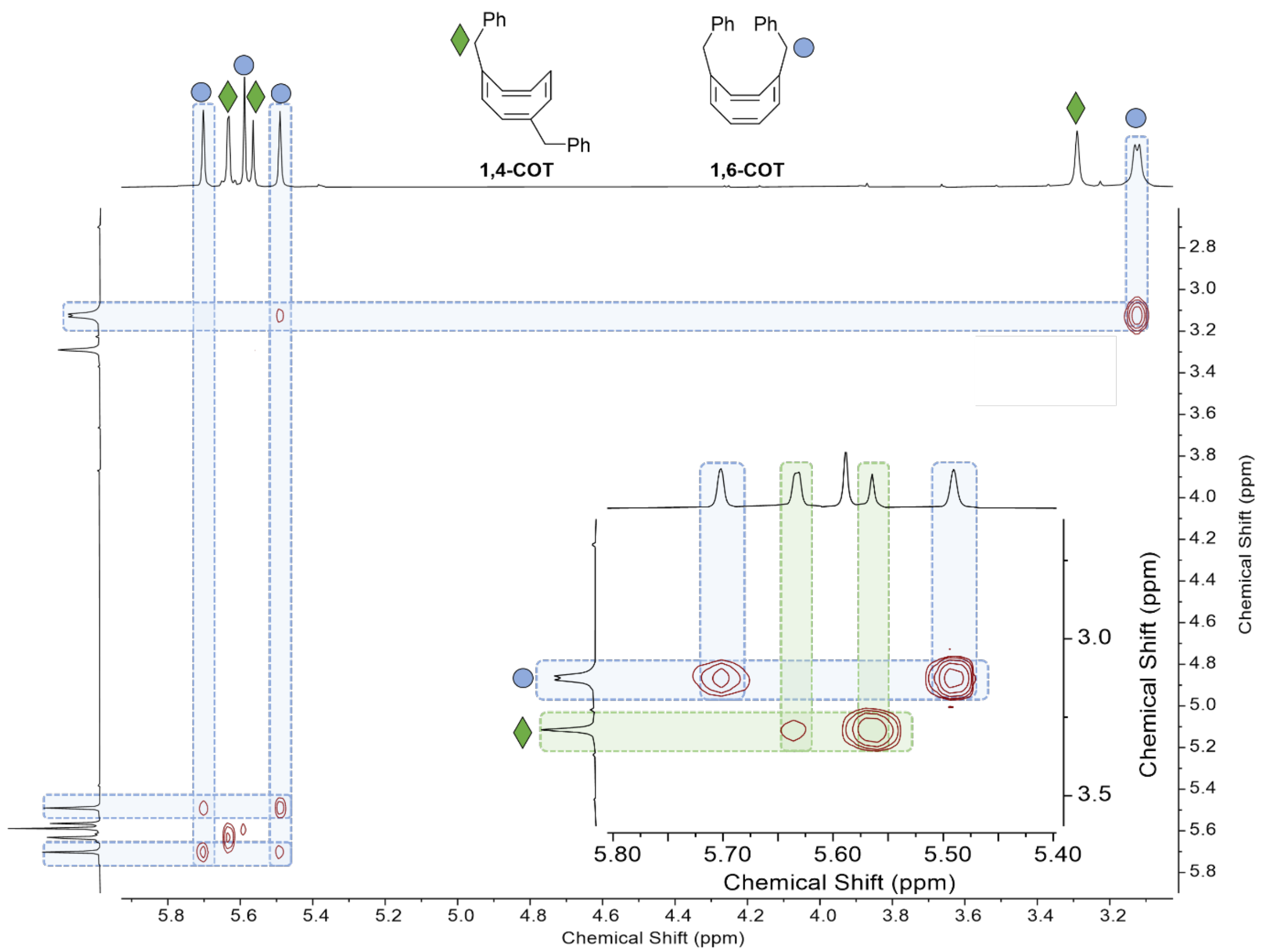

Figure S18: NOESY NMR experiment of **1,4/1,6-COT** in $CDCl_3$ to assign the corresponding protons.

2 Computational Evaluation

2.1 Equilibrium energies in solution

We investigated the conformers of **1,4-** and **1,6-COT** using Grimme's Conformer-Rotamer Ensemble Sampling Tool (CREST, version 3.0.1) at the GFN2 level of theory.[1,2] The conformational ensemble for each isomer was further optimized with Grimme's Commandline Energetic Sorting Tool (CENSO version 1.2.0)[3] at the ωB97X-D4/def2-TZVP + SMD + GmRRHO(GFN2[ALPB]-Bhess) // r2SCAN-3c[SMD] level of theory. This workflow employed XTB (version 6.7.0) and ORCA (version 5.0.4).[4,5] The settings of the CENSO configuration file used to compute the equilibrium energies of each isomer are provided below.

GENERAL SETTINGS:
nconf: all
charge: 0
unpaired: 0
solvent: gas
prog_rrho: xtb
temperature: 313.15
trange: [273.15, 378.15, 5]
multitemp: on
evaluate_rrho: on
consider_sym: on
bhess: on
imagthr: automatic
sthr: automatic
scale: automatic
rmsdbias: off
sm_rrho: alpb
progress: off
check: on
prog: orca
func: r2scan-3c
basis: automatic
maxthreads: 1
omp: 1
balance: off
cosmorsparam: automatic

$PART0 - CHEAP-PRESCREENING - SETTINGS:
part0: on
func0: b97-d3
basis0: def2-SV(P)
part0_gfnv: gfn2
part0_threshold: 4.0

$PART1 - PRESCREENING - SETTINGS:
func and basis is set under GENERAL SETTINGS
part1: on
smgsolv1: smd
part1_gfnv: gfn2
part1_threshold: 3.5

$PART2 - OPTIMIZATION - SETTINGS:
func and basis is set under GENERAL SETTINGS
part2: on
prog2opt: prog
part2_threshold: 2.5
sm2: smd
smgsolv2: smd
part2_gfnv: gfn2
ancopt: on
hlow: 0.01
opt_spearman: on
part2_P_threshold: 100
optlevel2: automatic
optcycles: 8
spearmanthr: -4.0
radsize: 10
crestcheck: off

$PART3 - REFINEMENT - SETTINGS:
part3: on
prog3: prog
func3: wb97x-d4
basis3: def2-TZVP

smgsolv3: smd

part3_gfnv: gfn2

part3_threshold: 95

Table S13: Computed energies for the ensemble of 1,6-COT and 1,4-COT using censo for a given solvent at 313 K.

**Solvent**	**Ensemble Gibbs free energy for 1,6-COT [Hartree]**	**Ensemble Gibbs free energy for 1,4-COT [Hartree]**	**Calculated $\Delta G_{1,6-1,4}$ [kcal mol^{-1}]**
**MeOH-*d*$_4$**	-850.1267337	-850.1260333	-0.44
**n-Hexane-*d*$_{14}$**	-850.1274845	-850.1264933	-0.62
**MeCN-*d*$_3$**	-850.1305799	-850.1302281	-0.22
**Acetone-*d*$_6$**	-850.1309731	-850.1305468	-0.27
**Cyclohexane-*d*$_{12}$**	-850.1269551	-850.1258667	-0.68
**THF-*d*$_8$**	-850.1291150	-850.1284149	-0.44
**DCE-*d*$_4$**	-850.1321860	-850.1315090	-0.42
**Toluol-*d*$_8$**	-850.1294881	-850.1282161	-0.80
**$CDCl_3$**	-850.1327711	-850.1319602	-0.51
**Benzene-*d*$_6$**	-850.1295419	-850.1285415	-0.63
**DMSO-*d*$_6$**	-850.1237126	-850.1229253	-0.49
**CS_2**	-850.1297982	-850.1287390	-0.66

2.2 Thermodynamic data and coordinates of TT-1,6-COT and PD-1,6-COT.

To further analyse the arrangements of **1,4-/1,6-COT**, the most stable **1,4-COT** structure, as well as the most stable TT-shaped and PD-shaped structures of **1,6- COT**, were extracted from the results of Section 2.1. These structures were further optimized at the ωB97X-D4/def2-TZVPP level of theory in the gas phase using ORCA (version 5.0.4).[4,5] Energies are given in detail for the isomers below. The solvent-accessible surface area (water) was calculated for the optimized geometries of TT-shaped 1,6-COT, PD-shaped 1,6-COT, and the most stable 1,4-COT. These computations were performed in ORCA at ωB97x-d4/def2-TZVPP level of theory,[6,7] using the same optimized geometries.

Table S14: Computed Energies in hartree at the ωB97x-d4 def2-TZVPP level of theory for the most stable COT species in gas Phase.

	**TT-1,6-COT**	**PD-1,6-COT**	**1,4-COT**
Electronic energy	-851.06037	-851.05720	-851.05643
Zero point energy	0.35549	0.35595	0.35532
E_{el} +ZPVE	-850.70488	-850.70126	-850.70111
Thermal vibrational correction	0.01548	0.01527	0.01563
Thermal rotational correction	0.00142	0.00142	0.00142
Thermal translational correction	0.00142	0.00142	0.00142
Total Enthalpy	-850.68562	-850.68221	-850.68171
Vibrational entropy	0.02730	0.02644	0.02766
Rotational entropy	0.01628	0.01611	0.01662
Translational entropy	0.02035	0.02035	0.02035
Final Gibbs free energy	-850.74956	-850.74511	-850.74634
Final Gibbs free energy (313K)	-850.75263	-850.74815	-850.74944

Table S15: Computed Energies in hartree at the ωB97x-d4 def2-TZVPP level of theory for benzene in gas Phase.

	**TT-Bn_2**	**PD-Bn_2**	**Monomer**
Electronic energy	-464.89369	-464.89337	-232.44436
Zero point energy	0.20340	0.20296	0.10116
E_{el} +ZPVE	-464.69029	-464.69041	-232.34320
Thermal vibrational correction	0.00788	0.00775	0.00153
Thermal rotational correction	0.00142	0.00142	0.00142
Thermal translational correction	0.00142	0.00142	0.00142
Total Enthalpy	-464.67864	-464.67889	-232.33789
Vibrational entropy	0.01393	0.01366	0.00205
Rotational entropy	0.01481	0.01386	0.01219
Translational entropy	0.01950	0.01950	0.01852
Final Gibbs free energy	-464.72688	-464.72592	-232.37065

Table S16: Computed Energies in hartree at the ωB97x-d4 def2-TZVPP level of theory for the most stable COT species in DCE using SMD.

	**TT-1,6-COT**	**PD-1,6-COT**	**1,4-COT**
Electronic energy	-851.08704	-851.08416	-851.08447
Zero point energy	0.35511	0.35570	0.35499
E_{el} +ZPVE	-850.73193	-850.72846	-850.72949
Thermal vibrational correction	0.01381	0.01449	0.01392
Thermal rotational correction	0.00142	0.00142	0.00142
Thermal translational correction	0.00142	0.00142	0.00142
Total Enthalpy	-850.71435	-850.71019	-850.71179
Vibrational entropy	0.02391	0.02482	0.02399
Rotational entropy	0.01628	0.01611	0.01662
Translational entropy	0.02035	0.02035	0.02035
Final Gibbs free energy	-850.77489	-850.77148	-850.77275

Table S17: Computed Energies in hartree at the ωB97x-d4 def2-TZVPP level of theory for Benzene in DCE using SMD.

	**TT-Bn_2**	**PD-Bn_2**	**Monomer**
Electronic energy	-464.91097	-464.91134	-232.45382
Zero point energy	0.20322	0.20305	0.10120
E_{el} +ZPVE	-464.70775	-464.70829	-232.35263
Thermal vibrational correction	0.00798	0.00774	0.00153
Thermal rotational correction	0.00142	0.00142	0.00142
Thermal translational correction	0.00142	0.00142	0.00142
Total Enthalpy	-464.69598	-464.69677	-232.34732
Vibrational entropy	0.01423	0.01363	0.00205
Rotational entropy	0.01481	0.01386	0.01219
Translational entropy	0.01950	0.01950	0.01852
Final Gibbs free energy	-464.74453	-464.74376	-232.38008

Coordinates: TT 1,6-di-Bn-COT

6	-0.172066000	-1.286381000	-0.464067000
6	0.026977000	-1.136623000	0.993857000
6	1.199924000	-0.952118000	1.585654000
6	2.499426000	-0.843243000	0.886931000
6	3.039328000	-1.837050000	0.183726000
6	2.421359000	-3.151874000	-0.063461000
6	1.244293000	-3.345939000	-0.658362000
6	0.354750000	-2.287407000	-1.169710000
1	-0.876965000	-1.113976000	1.596732000
1	1.224697000	-0.782430000	2.660545000
1	0.086638000	-2.366628000	-2.222159000
1	0.936757000	-4.369443000	-0.861702000
1	4.033477000	-1.691597000	-0.236159000
1	3.019213000	-4.024124000	0.190819000
6	3.175861000	0.501177000	1.026532000
1	3.274528000	0.761583000	2.084785000
1	4.181066000	0.455085000	0.601764000
6	-1.063310000	-0.245939000	-1.103731000
1	-0.571275000	0.727100000	-1.021723000
1	-1.167233000	-0.469202000	-2.168788000
6	2.359279000	1.566406000	0.323448000
6	2.320669000	1.606837000	-1.068571000
6	1.593685000	2.479992000	1.038692000
6	1.546908000	2.548940000	-1.730361000
6	0.809591000	3.421636000	0.381051000
6	0.785180000	3.460122000	-1.006233000
1	2.896053000	0.883654000	-1.636941000
1	1.608255000	2.454055000	2.123309000
1	1.533194000	2.570389000	-2.814319000
1	0.219539000	4.127042000	0.955634000
1	0.176917000	4.194375000	-1.522044000
6	-2.429027000	-0.165667000	-0.458211000
6	-2.827414000	0.970684000	0.237321000
6	-3.304968000	-1.246635000	-0.534855000
6	-4.079418000	1.032006000	0.839794000
6	-4.556040000	-1.189333000	0.061664000

6	-4.947676000	-0.047131000	0.752272000
1	-2.147417000	1.813636000	0.307529000
1	-2.996751000	-2.142649000	-1.064446000
1	-4.376307000	1.925181000	1.378263000
1	-5.228108000	-2.037277000	-0.010068000
1	-5.924865000	-0.000807000	1.219148000

Final Gibbs free energy –850.74956 Eh

--

Magnitude (Debye) 0.40598

GEPOL Surface-area (Bohr²) 1869.4598

Coordinates: PD 1,6-di-Bn-COT

6	-0.278887000	-2.337412000	0.959055000
6	0.776016000	-1.694903000	1.771612000
6	1.855889000	-1.046353000	1.347458000
6	2.276852000	-0.802680000	-0.047078000
6	2.466249000	-1.779709000	-0.935991000
6	2.333269000	-3.228609000	-0.707925000
6	1.265705000	-3.888933000	-0.266097000
6	-0.041930000	-3.311807000	0.081438000
1	0.596494000	-1.723226000	2.845254000
1	2.492855000	-0.584981000	2.100547000
1	-0.900298000	-3.785098000	-0.392407000
1	1.326139000	-4.974005000	-0.218264000
1	2.872936000	-1.504418000	-1.907523000
1	3.204773000	-3.814501000	-0.993384000
6	2.686050000	0.619639000	-0.374491000
1	3.418896000	0.933601000	0.378711000
1	3.207174000	0.622289000	-1.333284000
6	-1.691184000	-1.896801000	1.258689000
1	-2.382942000	-2.679676000	0.933743000
1	-1.819540000	-1.798161000	2.341176000
6	1.582173000	1.656170000	-0.419250000
6	0.844011000	1.995235000	0.714409000
6	1.331849000	2.345658000	-1.601389000
6	-0.108956000	3.000592000	0.665904000
6	0.386913000	3.364283000	-1.651189000

6	-0.334074000	3.697038000	-0.514922000
1	1.015792000	1.470591000	1.645947000
1	1.897842000	2.094862000	-2.492909000
1	-0.681695000	3.240592000	1.554209000
1	0.218272000	3.897508000	-2.580305000
1	-1.075801000	4.486591000	-0.548902000
6	-2.104326000	-0.594699000	0.596866000
6	-3.050544000	0.222231000	1.209879000
6	-1.623123000	-0.233049000	-0.658478000
6	-3.530292000	1.359653000	0.573064000
6	-2.101652000	0.901487000	-1.297846000
6	-3.062713000	1.696852000	-0.689458000
1	-3.427049000	-0.042891000	2.193123000
1	-0.866586000	-0.843409000	-1.137302000
1	-4.271881000	1.980272000	1.063897000
1	-1.711121000	1.173054000	-2.271027000
1	-3.432080000	2.584465000	-1.190181000

Final Gibbs free energy –850.74511 Eh

Magnitude (Debye) 0.41486

GEPOL Surface-area (Bohr²) 1758.4966

Coordinates: 1,4-di-Bn-COT

6	-1.470916000	-0.252111000	-0.511181000
6	-0.946231000	0.989921000	-1.120421000
6	-0.187952000	1.903766000	-0.520240000
6	0.310066000	1.831163000	0.864355000
6	1.050681000	0.849113000	1.371792000
6	1.558390000	-0.332449000	0.639893000
6	0.774674000	-1.222779000	0.034493000
6	-0.697794000	-1.211366000	-0.005587000
1	-1.297480000	1.197800000	-2.129762000
1	0.047195000	2.816105000	-1.064226000
1	-1.175490000	-2.119487000	0.352420000
1	1.241965000	-2.093950000	-0.417033000
1	0.090599000	2.689769000	1.495337000
1	1.404329000	0.950821000	2.396487000
6	-2.984397000	-0.356938000	-0.576925000

1	-3.406429000	0.469044000	0.005540000
1	-3.292970000	-0.178971000	-1.612985000
6	3.069346000	-0.466280000	0.703196000
1	3.507586000	0.428641000	0.249005000
1	3.368206000	-0.441434000	1.757211000
6	-3.556173000	-1.663908000	-0.097091000
6	-3.771821000	-2.713389000	-0.985648000
6	-3.842551000	-1.861637000	1.250968000
6	-4.261619000	-3.934259000	-0.540202000
6	-4.329471000	-3.080985000	1.702973000
6	-4.540470000	-4.122286000	0.807623000
1	-3.551030000	-2.571874000	-2.038803000
1	-3.674328000	-1.051779000	1.953555000
1	-4.426060000	-4.740035000	-1.246825000
1	-4.545228000	-3.218720000	2.756542000
1	-4.923050000	-5.074079000	1.157963000
6	3.633635000	-1.697360000	0.046337000
6	3.788074000	-2.879184000	0.765564000
6	3.980465000	-1.686545000	-1.301744000
6	4.280337000	-4.024676000	0.154332000
6	4.471340000	-2.829354000	-1.919175000
6	4.623683000	-4.002832000	-1.191561000
1	3.516408000	-2.901850000	1.816097000
1	3.860019000	-0.772034000	-1.873508000
1	4.396680000	-4.936039000	0.730052000
1	4.737258000	-2.802978000	-2.969967000
1	5.010105000	-4.895309000	-1.670242000

Final Gibbs free energy –850.74634 Eh

Magnitude (Debye) 0.28551

GEPOL Surface-area (Bohr²) 2049.1951

2.3 Noncovalent interaction plots

NCI plots provide a visualization index based on the electron density (ED) and the reduced density gradient (RDG). RDG isosurfaces of van der Waals dimers were computed with NCIPLOT[8] and visualized using VMD[9] (version 1.9.3) and Multiwfn[10] (version 3.8). Isosurfaces (isovalue s = 0.5, ranging from ρ(r) = –0.05 a.u. to +0.05 a.u.) are color-coded to represent interaction types: red for strong repulsion, blue for strong attractive interactions, and green for weak non-covalent interactions (NCI).

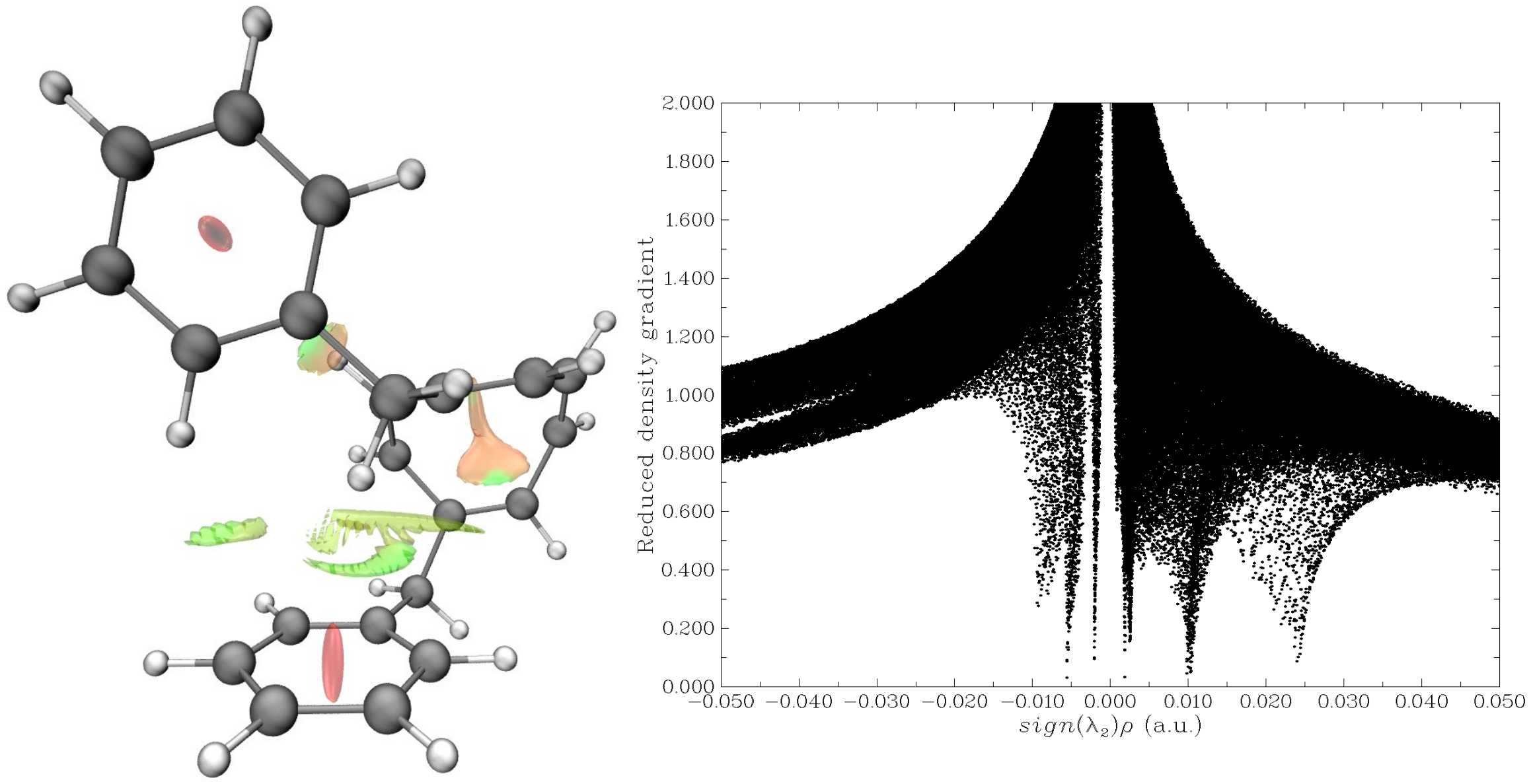

Figure S19: NCI plot and scatter plot of the most populated **TT-1,6-COT** geometry at the ωB97x-D4/def2-TZVPP level of theory.

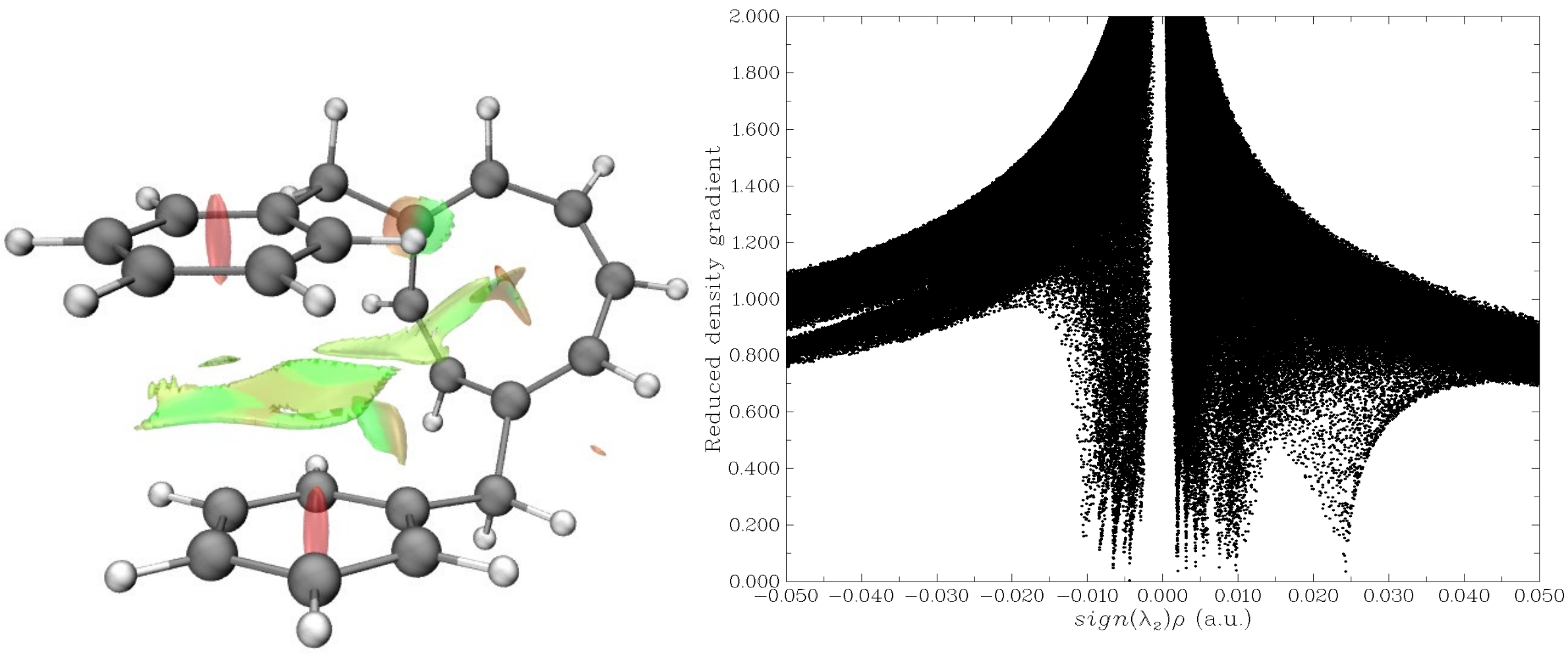

Figure S20: NCI plot and scatter plot of the most populated **PD-1,6-COT** geometry at the ωB97x-D4/def2-TZVPP level of theory.

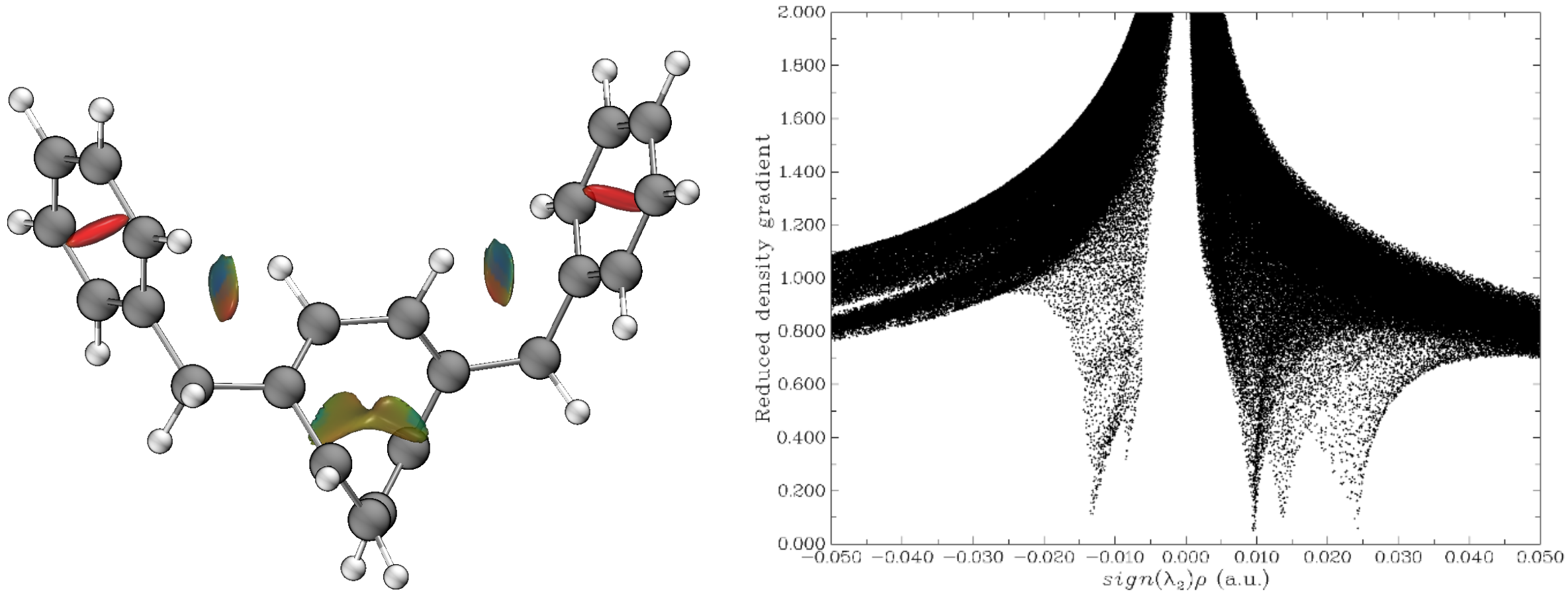

Figure S21: NCI plot and scatter plot of the most populated **1,4-COT** geometry at the ωB97X-D4/def2-TZVPP level of theory.

2.4 SAPT Analysis

With the aim to dissect the intramolecular interaction energy into its main contributors, we employed symmetry-adapted-perturbation theory (SAPT)[11] analysis as implemented in PSI4.[12] For accurate description of the energies, the ωB97X-D4/def2-TZVPP optimized geometries were analyzed with I-SAPT[13,14] method at fisapt0 jun-cc-pVDZ level of theory with the SIAO1 partitioning algorithm.[15] COT Backbone was defined as linker and the energies between the substituents was computed.

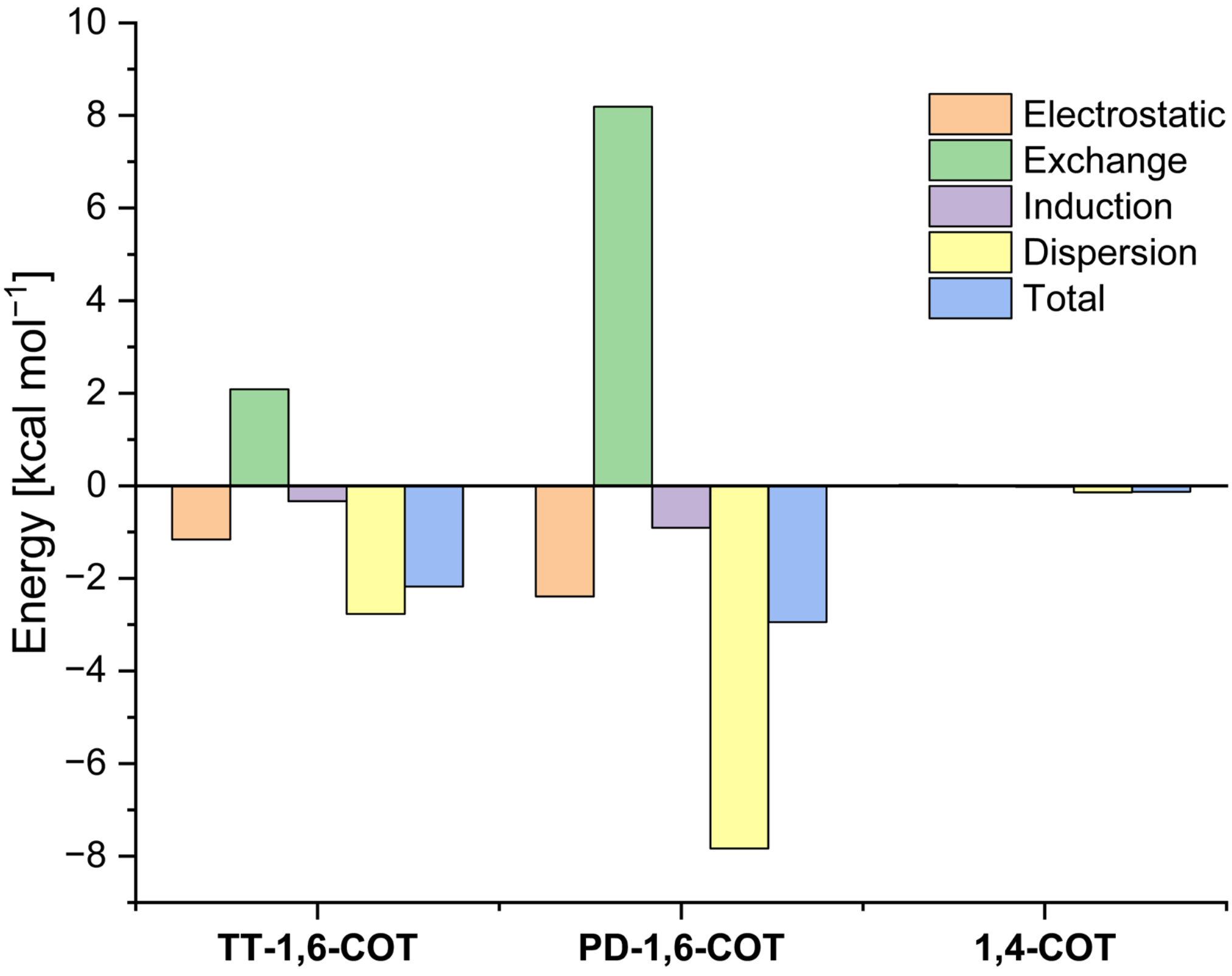

Figure S22: SAPT energies computed at fisapt0/jun-cc-pVDZ//ωB97x-d4/def2-TZVPP level of theory.

In a second approach, we focused on a distance dependent analysis of the aggregation for the TT and PD-conformation in a rigid dimer scan. For that, we removed the COT-linker and saturated the resulting radicals with hydrogen atoms. We varied the distance between both benzene moieties in small incremental steps of 0.2 Å. The stated distance corresponds to the distance of the benzene centers, which were measured with a dummy atom.

For the TT-conformation, the starting distance of 5.89 Å was obtained from the previous geometry optimizations. Interestingly the distance is larger than in the Benzene dimer (4.93. Å). Controversially, moving the residues closer together does not result in a stronger interaction energy, as the CH_2 spacing unit clashes into the opposing phenyl ring.

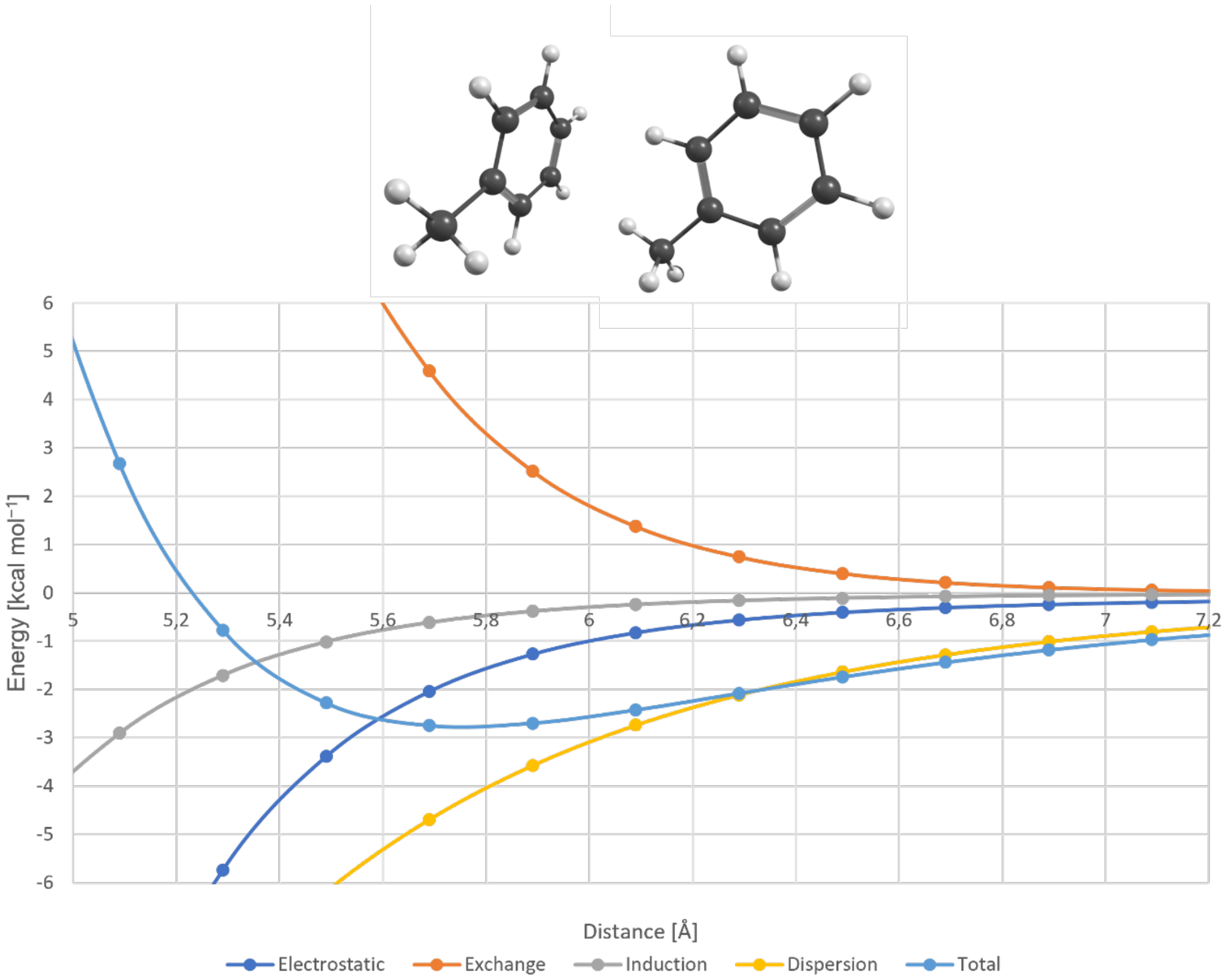

Figure S23: Rigid dimer scan of the TT-1,6-COT with removed COT-backbone.

The Phenyl groups in the PD-conformation are separated by 3.86 Å, which is very well in line with the literature value of 3.95 Å. Both, increasing and decreasing the Ph-Ph distance resulted in decreased interaction energy.

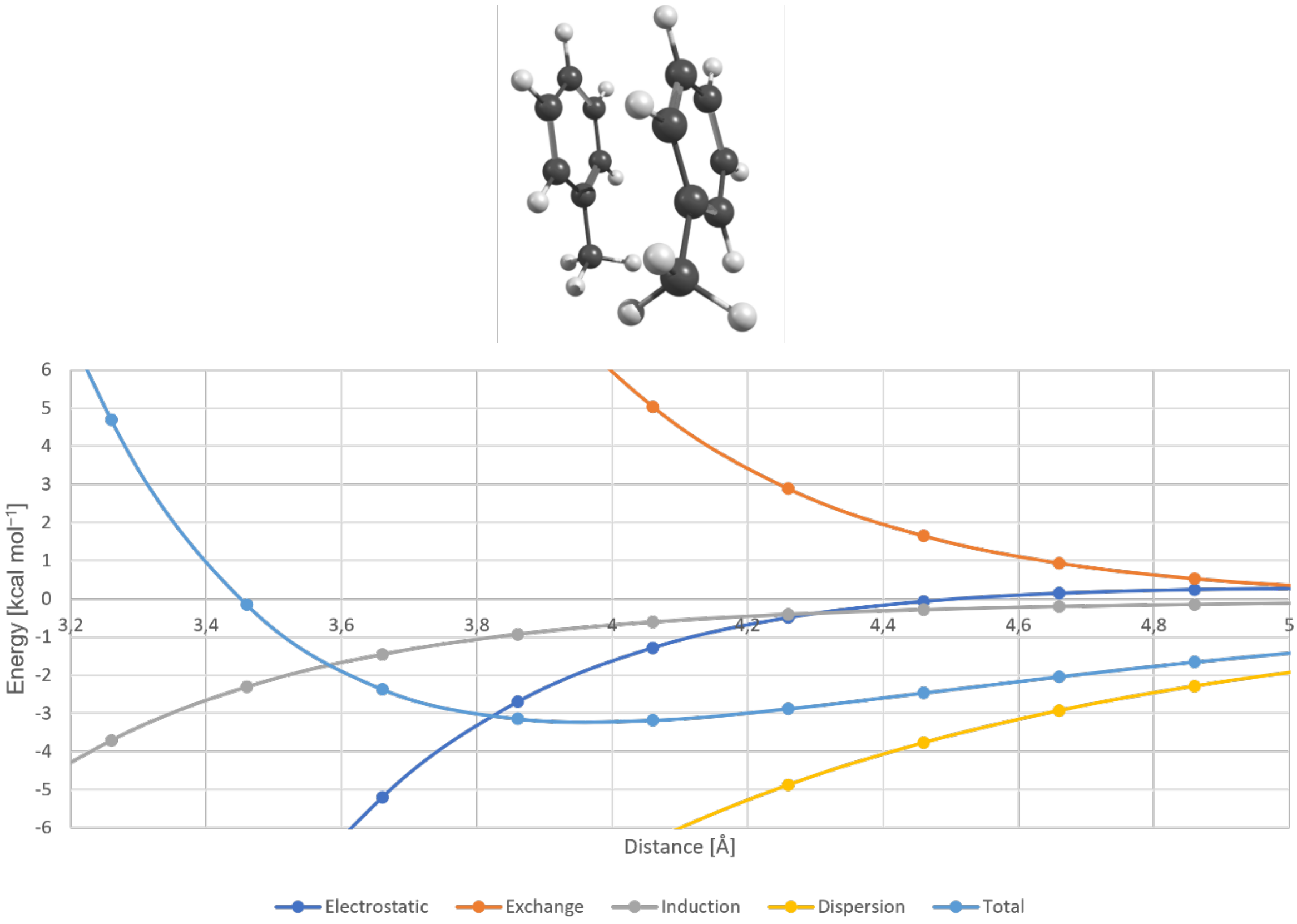

Figure S24: Rigid dimer scan of the PD-1,6-COT with removed COT-backbone.

2.5 Local Energy Decomposition (LED)

Additionally a Local Energy Decomposition analysis[16,17] was performed using ORCA (version 5.0.4).[4,5] The LED analysis[16,17] was performed at DLPNO−CCSD(T)/def2TZVP with tight natural orbital (TightPNO) settings with the ωB97x-d4/def2-TZVPP optimized geometries. Each molecule was split into three fragments (F1, F2, F3) as follows.

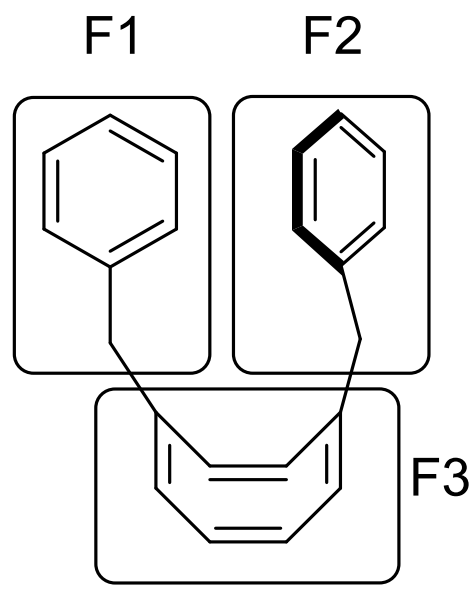

TT-1,6-COT

Table S18: Local Energy Decomposition of defined fragments F1, F2 and F3 for **TT-1,6-COT** at DLPNO−CCSD(T)/def2TZVP//ωB97x-d4/def2-TZVPP level of theory.

	**Interaction F1, F2 [kcal mol⁻¹]**	**Interaction F1, F3 [kcal mol⁻¹]**	**Interaction F2, F3 [kcal mol⁻¹]**
**Electrostatics**	67.12	-1305.27	-1315.40
**Exchange**	-1.42	-99.70	-101.08
**Dispersion (strong pairs)**	-5.46	-15.03	-17.85
**Dispersion (weak pairs)**	-0.54	-0.80	-0.80

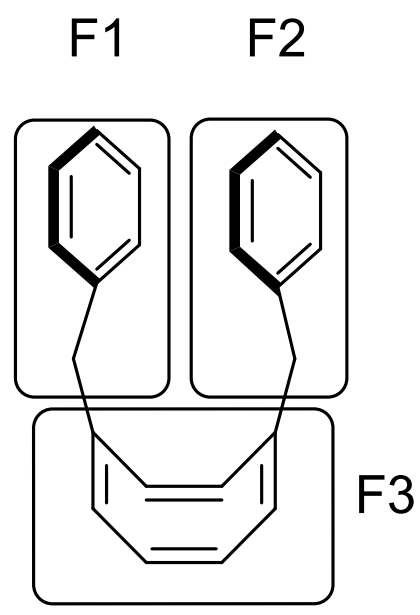

PD-1,6-COT

Table S19: Local Energy Decomposition of defined fragments F1, F2 and F3 for **PD-1,6-COT** at DLPNO−CCSD(T)/def2TZVP//ωB97x-d4/def2-TZVPP level of theory.

	**Interaction F1, F2 [kcal mol⁻¹]**	**Interaction F1, F3 [kcal mol⁻¹]**	**Interaction F2, F3 [kcal mol⁻¹]**
**Electrostatics**	60.92	-1312.56	-1304.05
**Exchange**	-4.42	-98.09	-98.91
**Dispersion (strong pairs)**	0.00	-15.31	-15.76
**Dispersion (weak pairs)**	-0.53	-0.78	-0.80

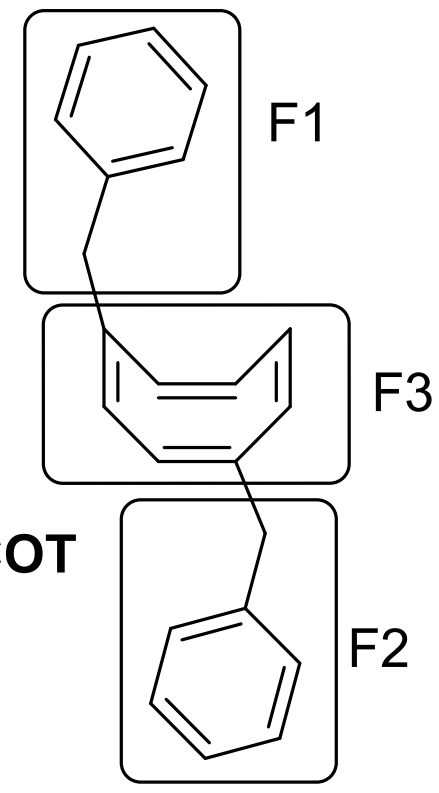

1,4-COT

Table S20: Local Energy Decomposition of defined fragments F1, F2 and F3 for **1,4-COT** at DLPNO−CCSD(T)/def2TZVP//ωB97x-d4/def2-TZVPP level of theory.

	**Interaction F1, F2 [kcal mol^{-1}]**	**Interaction F1, F3 [kcal mol^{-1}]**	**Interaction F2, F3 [kcal mol^{-1}]**
**Electrostatics**	59.57	-1301.72	-1301.64
**Exchange**	-0.03	-101.01	-101.03
**Dispersion (strong pairs)**	0.00	-15.76	-15.73
**Dispersion (weak pairs)**	-0.15	-0.82	-0.80

2.6 Computed ^{1}H and ^{13}C NMR Spectrum

Computed ^{1}H and ^{13}C-NMR spectra for the 1,6-isomers were obtained at the B3LYP-D3(BJ)/def2-TZVPP level of theory, using TMS as the reference for chemical shifts. The qualitative shifts observed for the benzylic positions in the ^{1}H and ^{13}C-NMR spectra align well with the experimentally measured shifts.

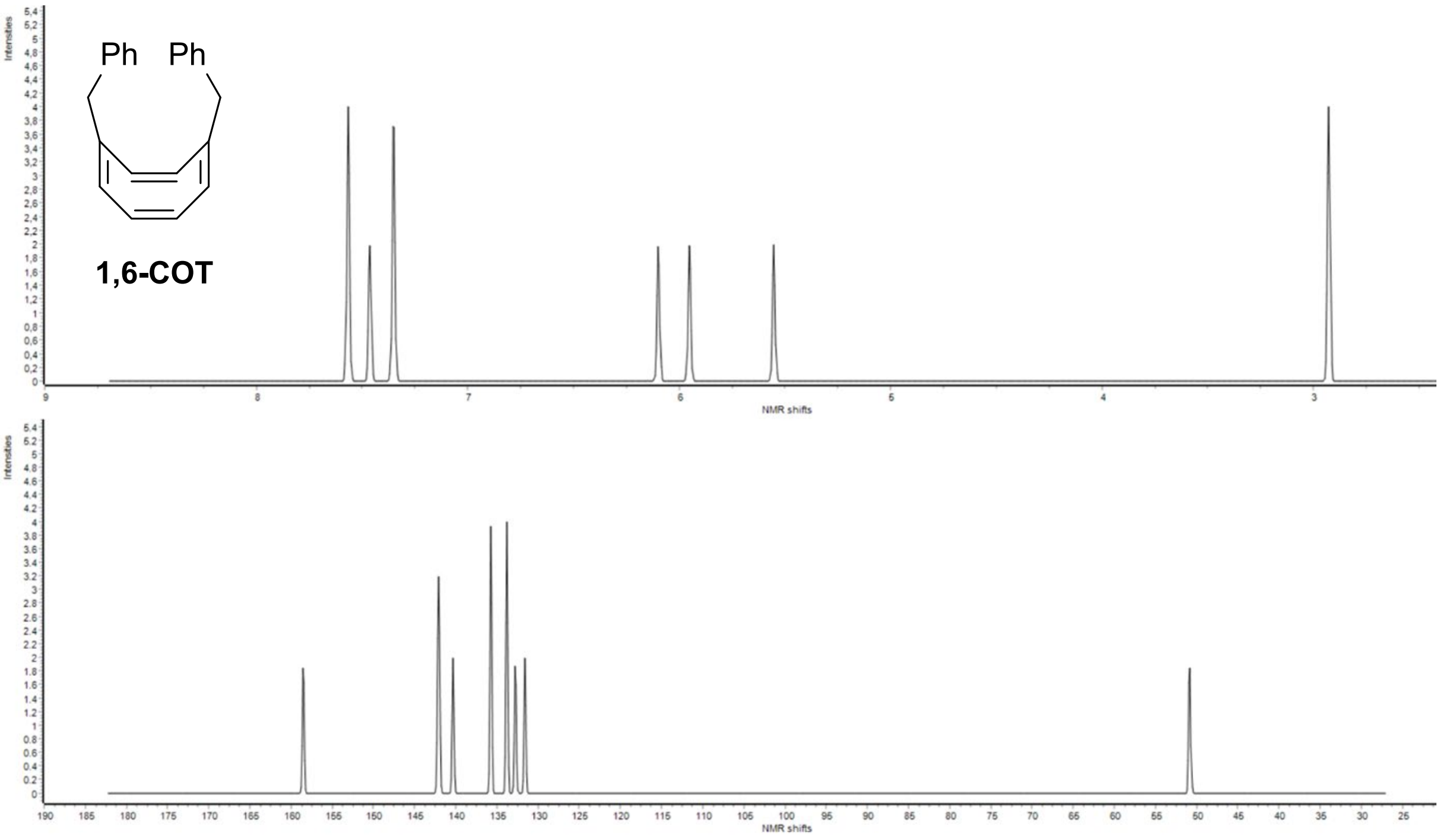

Figure S25: Computed ^{1}H and ^{13}C-NMR of TT-**1,6-COT** using TMS for reference shifts.

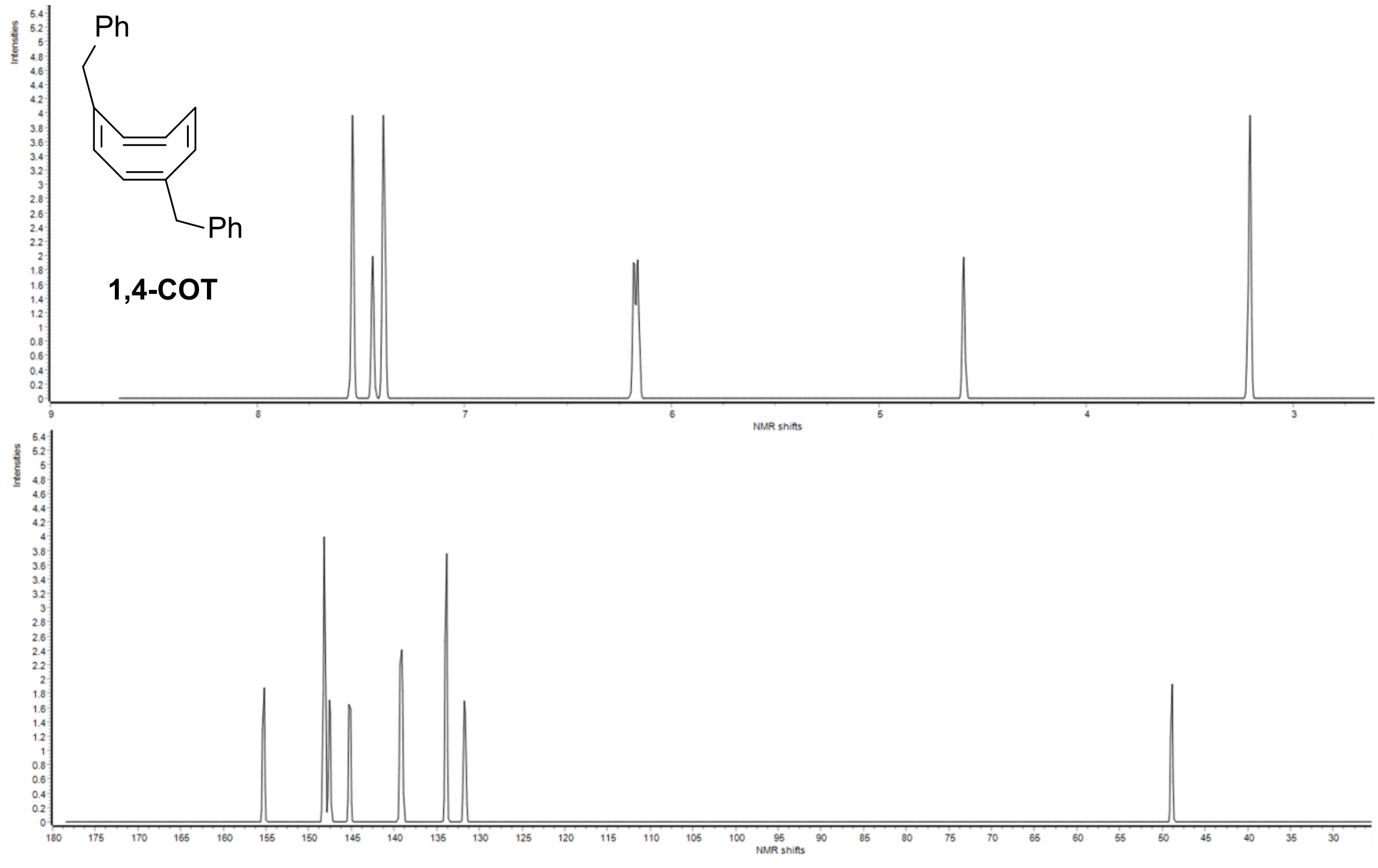

Figure S26: Computed ^{1}H and ^{13}C-NMR of **1,4-COT** using TMS for reference shifts.

3 Synthetic Details

3.1 General Information

Unless otherwise noted, chemicals were purchased from Acros Organics, TCI, Alfa Aesar, or Merck, at the highest purity grade available and were used without further purification. All solvents were distilled prior to use. All deuterated solvents for NMR measurement were purchased from abcr or deutero as ampoules (anhydrous 99 atom%-D analysis grade), but carbon tetrachloride and carbondisulfide, which were dried over 3 Å molecular sieves and distilled prior to use. As internal standard in carbon tetrachloride and carbondisulfide a glass capillary filled with deuterated benzene was added.

Thin Layer Chromatography (TLC) was performed on silica coated glass plates (Merck, silica 60 F254) with detection by UV–light (λ = 254 nm) and/or by staining with a with a cerium ammonium molybdate solution [CAM] followed by heat treatment. CAM–staining solution: cerium sulfate tetrahydrate (1.00 g), ammonium molybdate (25.0 g) and concentrated sulfuric acid (25.0 mL) in water (250 mL).

3.2 Synthetic procedures

5,8-Di-benzyl-cycloocta-1,3,6-triene **2**

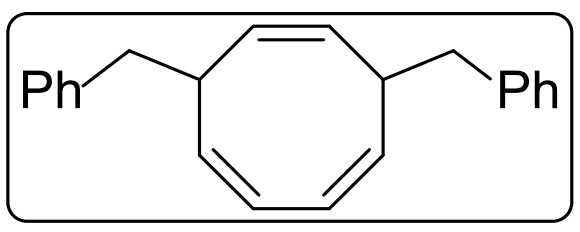

Cyclooctadiene (3.68 mL, 30.0 mmol, 1.00 equiv.) was dissolved in 38 mL of dry pentane. A solution of *n*-BuLi (2.5 M in *n*-hexane, 36.0 mL, 90.0 mmol, 3.00 equiv.) and TMEDA (13.5 mL, 90.0 mmol, 3.00 equiv.) were added dropwise 0 °C. The colorless solution was stirred at 0 °C for 15 min. Afterwards, the cooling bath was removed, and stirring was continued for 48 h at room temperature. DME (75 mL) was added and stirring was continued for another 15 min at room temperature. The mixture was filtered, and the deep red filtrate was cooled to −50 °C and treated with benzyl bromide (7.80 mL, 66.0 mmol, 2.20 equiv.). After 30 min at −50 °C, the reaction was quenched with saturated $NaHCO_3$ (100 mL) and extracted with *n*-hexane (3 x 100 mL). The combined organic phases were dried over anhydrous $MgSO_4$ and concentrated under reduced pressure to yield the crude product as yellow oil. The crude product was purified via column chromatography (silica gel, *n*-hexane:ethyl acetate 10:1) to yield triene **2** as a yellow oil. Because of its instability, triene **2** was used immediately in the next step.[18–21]

^{1}H NMR (400 MHz, CD_2Cl_2): [ppm] = 7.30−7.10 (m, 10H), 6.10−6.05 (m, 1H), 5.78−5.72 (m, 1H), 5.55−5.44 (m, 2H), 5.25−5.17 (m, 1H), 3.38 (s, 1H), 3.23−3.20 (m, 1H), 2.95−2.70 (m, 4H).

APCI: m/z: [M+H$^+$] calcd for $C_{22}H_{22}$, 287.1794; found, 287.1792.

R_f-value (*n*-hexane:ethyl acetate 10:1): 0.70

1,4/1,6-Di(benzyl)cyclooctatetraene **3**

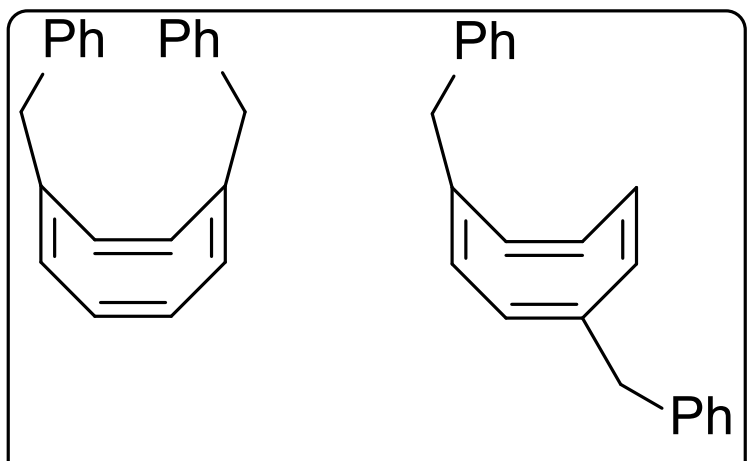

1,6-COT **1,4-COT**

Triene **2** (5.33 g, 18.6 mmol, 1.00 equiv.) was dissolved in 75 mL of dry THF. Afterwards, *n*-BuLi (2.5 M in *n*-hexane, 16.5 mL 40.9 mmol, 2.20 equiv.) was added dropwise at −50 °C over 15 min. The reaction mixture was stirred for 90 min at 0 °C. The reaction mixture was again cooled to −50 °C and iodine (5.20 g, 20.5 mmol, 1.10 equiv.) was added portionwise. After 16 h at room temperature, the reaction was quenched with saturated Na_2SO_3 (100 mL) and extracted with *n*-hexane (3 x 100 mL). The combined organic layers were dried over anhydrous $MgSO_4$ and concentrated under reduced pressure to yield the crude product as a brown oil. The product was purified via column chromatography (silica gel, *n*-hexane) to yield **1,4/1,6-COT** (4.56 g, 16.1 mmol, 54% over two steps) as a yellow oil. Further purity was achieved via $AgNO_3$-impregnated silica chromatography (*n*-hexane:DCM 50:1) and RP HPLC.[18–21]

HPLC (Eurospher II $C_{18}H$; 1mL/min, acetonitrile, 254 nm): 4.61 min, 2034.4 mAU, 400.8 mAU min.

^{1}H NMR (400 MHz, $CDCl_3$): [ppm] = 7.29−7.24 (m, 8H), 7.21−7.15 (m, 12H), 5.77 (s, 2H), 5.70−5.69 (m, 2H), 5.66 (s, 2H), 5.63 (s, 1H), 5.56 (s, 2H), 3.36 (s, 2H), 3.19 (s, 4H).

^{13}C NMR (400 MHz, $CDCl_3$): [ppm] = 143.6, 142.4, 139.6, 139.5, 133.9, 133.3, 131.6, 131.4, 129.2, 129.0, 128.6, 128.4, 128.3, 127.6, 126.3,126.3, 44.1, 43.9.

IR (ATR): $\tilde{\nu}$ [cm^{-1}] = 3024.9 (w, aromatic υ_{CH}), 2942.7 (w, aliphatic υ_{CH}), 1640.5 (w, $\upsilon_{C=C}$), 1601.3 (w, ring vibration), 1493.6, 1452.3 (m, δ_{CH_2}), 738.4, 715.7 (s, aromatic δ_{CH}).

APCI: m/z: [M+H$^+$] calcd for $C_{22}H_{20}$, 285.1638; found, 285.1637.

Rf-value (*n*-hexane): 0.14

3.3 NMR Spectra

5,8-Di(benzyl)cycloocta-1,3,6-triene **2**

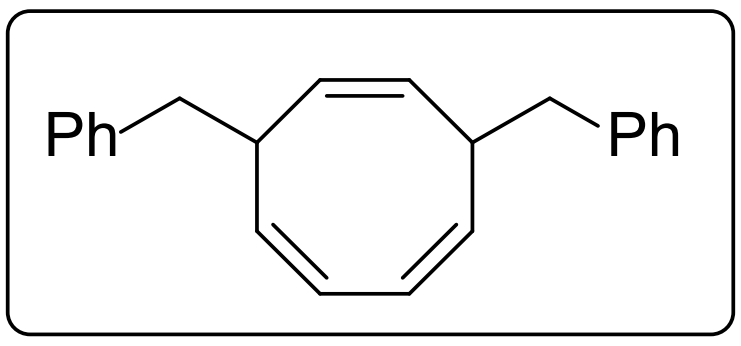

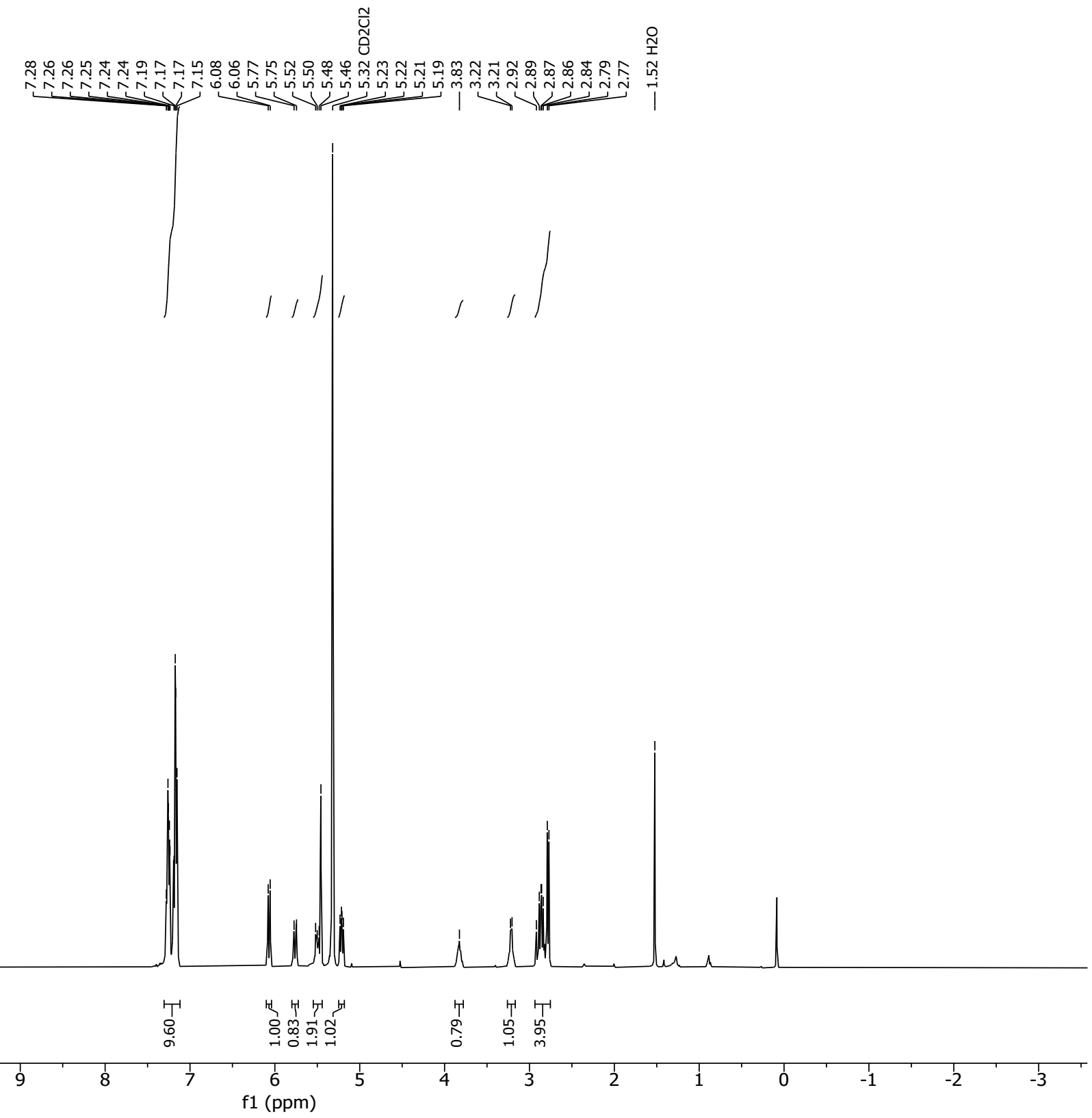

1,4/1,6-Di(benzyl)cyclooctatetraene **3**

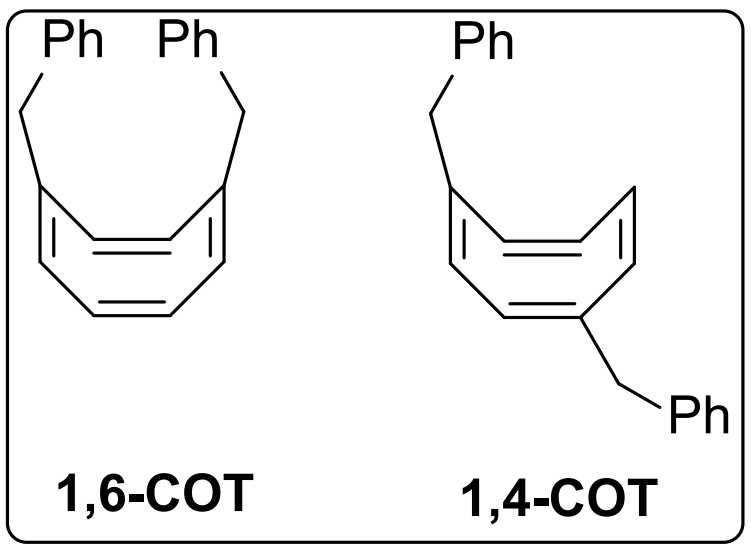

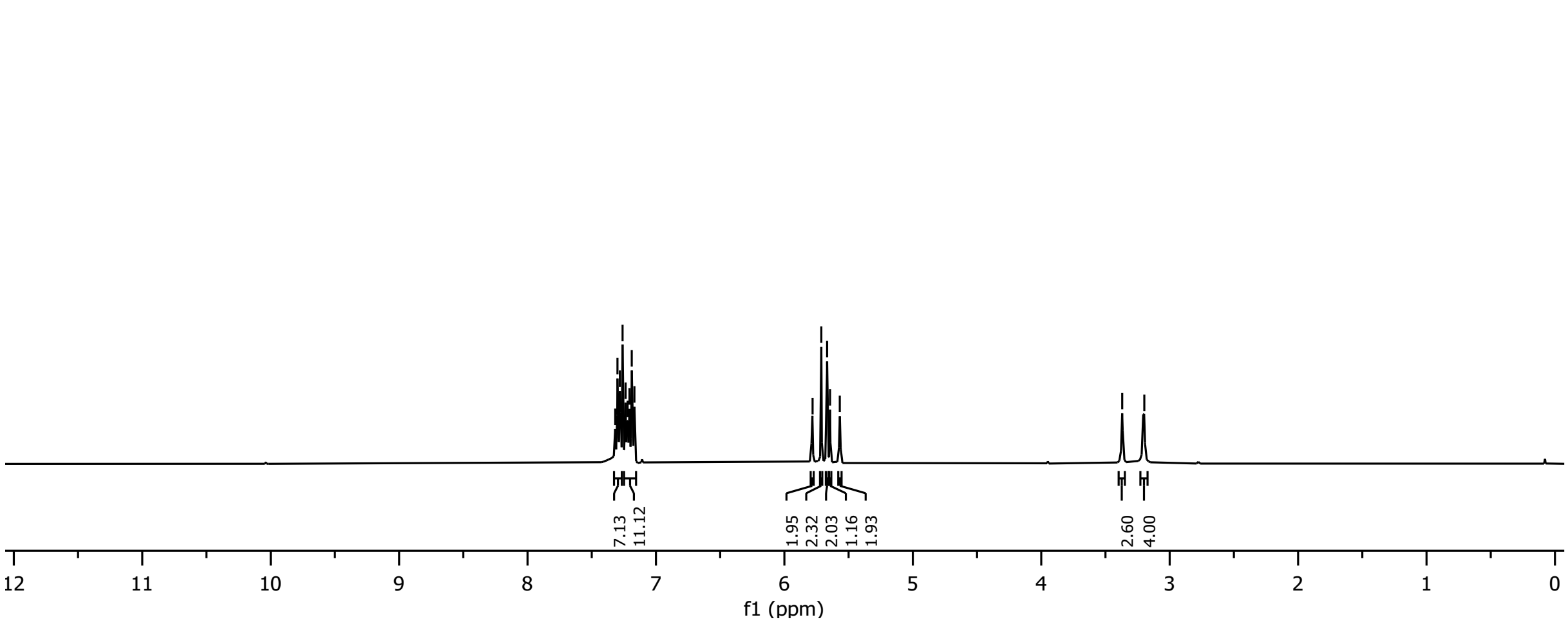

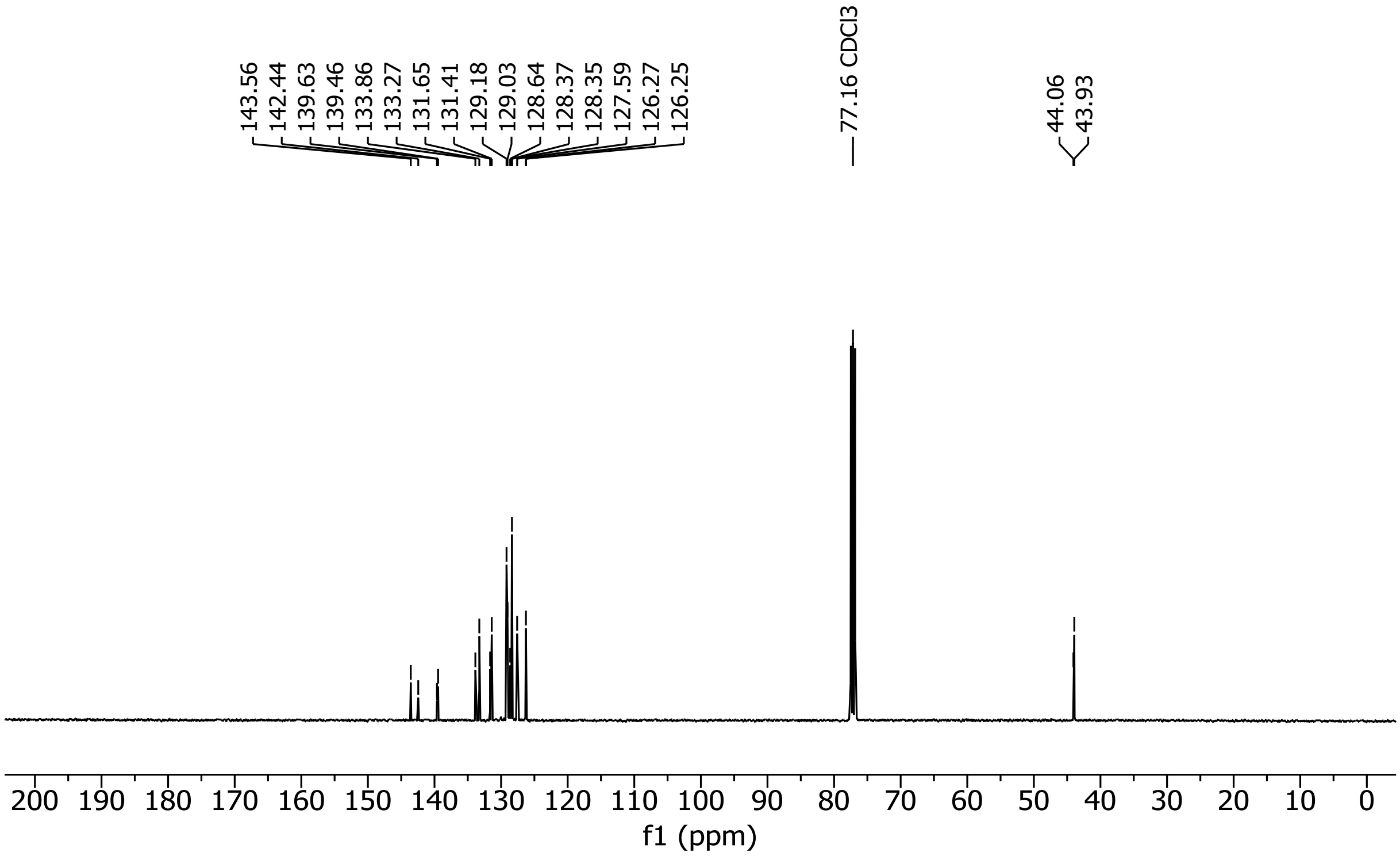

4 References

[1] P. Pracht, F. Bohle, S. Grimme *Phys. Chem. Chem. Phys.* **2020**, *22*, 7169–7192.

[2] P. Pracht, S. Grimme, C. Bannwarth, F. Bohle, S. Ehlert, G. Feldmann, J. Gorges, M. Müller, T. Neudecker, C. Plett, S. Spicher, P. Steinbach, P. A. Wesołowski, F. Zeller *J. Chem. Phys.* **2024**, *160*, 114110.

[3] S. Grimme, F. Bohle, A. Hansen, P. Pracht, S. Spicher, M. Stahn *J. Phys. Chem. A* **2021**, *125*, 4039–4054.

[4] F. Neese *WIREs Comput. Mol. Sci.* **2012**, *2*, 73–78.

[5] F. Neese *WIREs Comput. Mol. Sci.* **2022**, *12*, e1606.

[6] J.-D. Chai, M. Head-Gordon *Phys. Chem. Chem. Phys.* **2008**, *10*, 6615.

[7] F. Weigend, R. Ahlrichs *Phys. Chem. Chem. Phys.* **2005**, *7*, 3297.

[8] E. R. Johnson, S. Keinan, P. Mori-Sánchez, J. Contreras-García, A. J. Cohen, W. Yang *J. Am. Chem. Soc.* **2010**, *132*, 6498–6506.

[9] W. Humphrey, A. Dalke, K. Schulten *J. Mol. Graph.* **1996**, *14*, 33–38.

[10] T. Lu, F. Chen *J. Comput. Chem* **2012**, *33*, 580–592.

[11] K. Szalewicz *WIREs Comput. Mol. Sci.* **2012**, *2*, 254–272.

[12] J. M. Turney, A. C. Simmonett, R. M. Parrish, E. G. Hohenstein, F. A. Evangelista, J. T. Fermann, B. J. Mintz, L. A. Burns, J. J. Wilke, M. L. Abrams, N. J. Russ, M. L. Leininger, C. L. Janssen, E. T. Seidl, W. D. Allen, H. F. Schaefer, R. A. King, E. F. Valeev, C. D. Sherrill, T. D. Crawford *WIREs Comput. Mol. Sci.* **2012**, *2*, 556–565.

[13] R. M. Parrish, J. F. Gonthier, C. Corminbœuf, C. D. Sherrill *J. Chem. Phys.* **2015**, *143*, 051103.

[14] E. Pastorczak, A. Prlj, J. F. Gonthier, C. Corminboeuf *The Journal of Chemical Physics* **2015**, *143*, 224107.

[15] D. Luu, K. Patkowski *J. Phys. Chem. A* **2023**, *127*, 356–377.

[16] W. B. Schneider, G. Bistoni, M. Sparta, M. Saitow, C. Riplinger, A. A. Auer, F. Neese *J. Chem. Theory Comput.* **2016**, *12*, 4778–4792.

[17] A. Altun, M. Saitow, F. Neese, G. Bistoni *J. Chem. Theory Comput.* **2019**, *15*, 1616–1632.

[18] H. F. König, L. Rummel, H. Hausmann, J. Becker, J. M. Schümann, P. R. Schreiner *J. Org. Chem.* **2022**, *87*, 4670–4679.

[19] N. C. Burton, F. G. N. Cloke, S. C. P. Joseph, H. Karamallakis, A. A. Sameh *J. Organomet. Chem.* **1993**, *462*, 39–43.

[20] J. S. Parry, Cloke, S. J. Coles, M. B. Hursthouse *J. Am. Chem. Soc.* **1999**, *121*, 6867–6871.

[21] O. T. Summerscales, F. G. N. Cloke, P. B. Hitchcock, J. C. Green, N. Hazari *J. Am. Chem. Soc.* **2006**, *128*, 9602–9603.
